# The Wild Rice Locus *CTS-12* Mediates ABA-Dependent Stomatal Opening Modulation to Limit Water Loss Under Severe Chilling Stress

**DOI:** 10.3389/fpls.2020.575699

**Published:** 2020-10-30

**Authors:** Weijian Cen, Wenlong Zhao, Mingqing Ma, Siyuan Lu, Jianbin Liu, Yaqi Cao, Zhenhua Zeng, Hanxing Wei, Shaokui Wang, Rongbai Li, Jijing Luo

**Affiliations:** ^1^College of Life Science and Technology State Key Laboratory for Conservation and Utilization of Subtropical Agro-bioresources, Guangxi University, Nanning, China; ^2^College of Agriculture, Guangxi University, Nanning, China; ^3^College of Agriculture, South China Agricultural University, Guangzhou, China

**Keywords:** wild rice (*Oryza rufipogon* Griff), chilling tolerance, O2PLS/OPLS-DA, transcriptomic profiling, metabolic profiling, ABA, stomatal modulation, water uptake/loss balance

## Abstract

A near-isogenic line (NIL) DC90 which was generated by introgressing a wild rice (*Oryza rufipogon* Griff.) locus *CTS-12* into the 9311(*Oryza sativa* L. ssp. *indica*) background confers chilling tolerance phenotype. Here, our pilot trials showed that chilling tolerance was positively correlated with abscisic acid (ABA) biosynthesis. To understand how *CTS-12* mediated the ABA-dependent multi-levels of regulation, the integration of transcriptomic and metabolomic profiling using the two-way orthogonal projections to latent structures (O2PLS) and discriminant analysis (OPLS-DA) modeling was performed to investigate the mechanisms underlying chilling tolerance. Our results revealed that metabolic shifts, including the activation of stachyose biosynthesis, amino acid metabolism pathways, phenylpropanoid/flavonoid biosynthesis, ABA biosynthesis, and perturbation of glycolysis, occurred under chilling treatment; in the recovery period, glutamate-related pathways, β-alanine biosynthesis and degradation, and serotonin biosynthesis pathways were differentiated between 9311 and DC90. Particularly, the differentially accumulated metabolites (DAMs) and differentially expressed genes (DEGs), including galactinol, β-alanine, glutamate, naringenin, serotonin, ABA, and *LOC_Os03g44380* (9-*cis*-epoxycarotenoid dioxygenase 3, *OsNCED3*), might be involved in the chilling tolerance variation of 9311 and DC90. CRISPR/Cas9-edited *OsNCED3* resulted in chilling sensitive of *japonica* rice ZH11, demonstrating the involvement of ABA pathway in chilling stress response. In addition, chilling tolerance of rice was associated with the balance of water uptake and loss that was modulated by stomatal movement under chilling stress. Therefore, we speculated that the *CTS-12*-mediated ABA signaling pathway leads to transcriptional regulation of chilling-responsive genes and, in turn, triggers metabolic shifts to coordinately regulate the stomatal movement of guard cells. The results of this study improve our understanding of the multilevel regulation of wild rice in response to chilling stress.

## Introduction

Higher plants are sessile and usually adopt the ‘overcome’ strategy upon encountering any extreme environmental stresses, in contrast to animals, which preferentially opt for the avoidance of unfavorable circumstances. Chilling stress (above 0°C) is a wide-spread factor with deleterious effects on several facets of plant life and causes substantial economic losses in agriculture (Pareek et al., 2017). Irreversible biochemical and physiological perturbations imposed by chilling stress cause visible injury to plants, such as leaf discoloration and wilting, accelerated aging, incomplete ripening, and even death (Zhang et al., 2016). The mechanisms underlying chilling responses upon exposure to chilling stress are well established, and many genes that are either up- or down-regulated have been identified (Seki et al., 2001; Fowler and Thomashow, 2002). The *ICE1* (*Inducer of CBF expression 1*)-*CBFs* (*C-repeat-binding factors*)-*COR* (*Cold-responsive*) regulon is a well-characterized cold-responsive signaling pathway (Thomashow, 1999; Chinnusamy et al., 2006). In addition, ABA also plays a vital role in chilling stress signaling cascades. Chilling stress induces the accumulation of endogenous ABA in plants (Chawade et al., 2013; Sah et al., 2016). Likewise, exogenous application of ABA can also enhance the chilling tolerance of plants (Xing and Rajashekar, 2001). Recent advances in our understanding of ABA signal transduction suggest that three protein classes, pyracbactin resistance/pyracbactin resistance-like/regulatory component of ABA receptor (PYR/PYL/RCARs), are ABA receptors. In this signaling pathway, protein phosphatase 2Cs (PP2Cs) and sucrose non-fermenting 1 (SNF1)-related protein kinase 2s (SnRK2s), in particular, open stomata 1 (OST1)/SnRK2.6, act as negative and positive regulators, respectively (Yoshida et al., 2006; Park et al., 2009; Umezawa et al., 2009). In the absence of ABA, SnRK2s are inhibited by PP2Cs, while plants exposed to abiotic stresses, increased ABA levels lead to formation of the PYR/PYL/RCAR-PP2C complex (Fujii et al., 2009; Ma et al., 2009; Park et al., 2009) and release of SnRK2s inhibition. The activation of SnRK2s further regulates the transcription of ABA-responsive genes and ion channels on cell membranes (Nishimura et al., 2009). The activation of S-/R-type anion channels slow anion channel-associated 1 (SLAC1) and quickly activating anion channel 1 (QUAC1) on the membranes by phosphorylation results in the efflux of anion from guard cells and a reduced cell turgor, thereby promoting stomatal closure (Assmann and Jegla, 2016; Hedrich and Geiger, 2017). In the ABA-mediated stomatal movement signaling pathway, ROS, NO, and Ca^2+^ act as a key convergence point for the regulation of stomatal closure. The increased levels of ROS, NO, or Ca^2+^ in guard cells trigger multiple events in either downstream or upstream processes (Agurla and Raghavendra, 2016).

However, multiple levels of regulation are involved in the response of plants to chilling stress (Zhang et al., 2016). Therefore, a comprehensive understanding of the multiple levels of plant bioprocesses, including transcriptomic, proteomic, and metabolomic reprogramming, is needed to cope with the challenges of adverse environmental stresses. Currently, the growing interest in linking multiomics data could, in turn, facilitate an overview of almost all bioprocesses of plants involved in stress responses that individual studies alone would otherwise not achieve (Copley et al., 2017). For efficient extraction of informative biological mechanisms from the analyses based on data from different omics platforms, it is necessary to develop methods to allow relevant biological processes to be easily described and interpreted. O2PLS, a supervised multivariate analysis, is an algorithm that can integrate data from different omics platforms such as RNA-Seq and LC-MS/MS; it is also capable of multiblock bidirectional correlations (Trygg and Wold, 2003; Kirwan et al., 2012). By the modeling of O2PLS, one could identify joint variations between two datasets, respectively, as well as systematic variations that are unique to each dataset (Bylesjo et al., 2007). Analyses of joint variation and unique variables help us predict the occurrence of biological responses in cells (Trygg and Wold, 2003; Bylesjo et al., 2007). OPLS-DA can be used to identify differential variables between classes (Bylesjö et al., 2006). Thus far, although the application of O2PLS in integrating plant transcript and metabolite profiling data can be found in recent literature (Szymanski et al., 2014; Copley et al., 2017), reports using O2PLS to explore the functional links between different omics levels in plants in response to chilling stress are rare.

Changes in metabolites are considered as the ultimate responses of plants to adverse stresses; thus, metabolites are the real players in plant stress responses (Zhang et al., 2016). Therefore, metabolic responses to chilling stress in plants have attracted increasing attention. In a recent study, the accumulation of galactinol and raffinose in plants under abiotic stresses has suggested their possible roles in ROS scavenging (Nishizawa et al., 2008). In addition, amino acid metabolisms are also perturbed under chilling stress, with a marked accumulation of some amino acids, such as glutamate, ornithine, proline, cysteine, and polyamine, and a decline in others, such as branched-chain amino acids (Kaplan et al., 2007; Usadel et al., 2008). In Arabidopsis (*A. thaliana*), the biosynthesis of flavonoids, anthocyanins, and phenylpropanoids is induced by chilling stress; of which, anthocyanin is positively correlated with chilling tolerance in some ecotypes, such as Tenela (Te) and C24 (Marczak et al., 2008). Thus far, despite recent advances in the studies of plant responses to chilling stress, relatively little has been reported based on the integration of ‘omics’ data from two platforms. Therefore, an exploration of the functional links between different omics levels will shed new light on identification of the mechanisms underlying chilling stress tolerance in plants.

In this study, we aimed to infer the potential mechanisms relevant to chilling stress tolerance of wild rice. DC90, a previously developed chromosome segment substitution line (CSSL) which harbors a chilling-tolerant wild rice locus *CTS-12* (Wang et al., 2017), and its recurrent parent 9311 (chilling sensitive variety) were used to perform non-targeted metabolomic and transcriptomic profilings under chilling and recovery treatment to investigate the impact of *CTS-12* on the multilevel chilling-induced responses of plants that differentiate the chilling tolerance capacity between the two genotypes. O2PLS and OPLS-DA modeling were performed to integrate two datasets to identify joint variations between them. Our results suggest that the DAMs and DEGs, including galactinol, β-alanine, glutamate, naringenin, serotonin, ABA, and *LOC_Os03g44380*, might be involved in the chilling tolerance variation of 9311 and DC90.

## Materials and Methods

### Plant Materials, Growth, and Experimental Treatment

**FIGURE 1:**
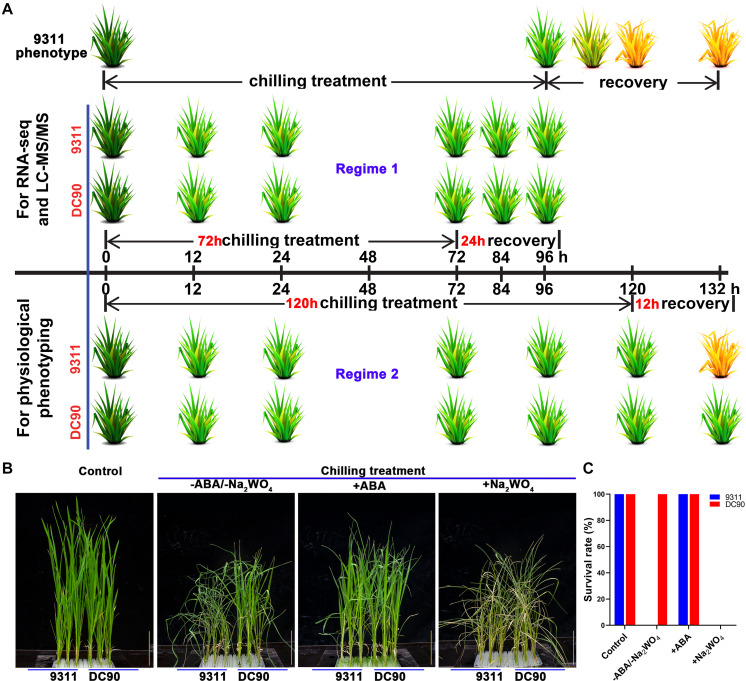
Chilling stress phenotypes of 9311 and DC90 in response to exogenous application of ABA and its biosynthetic inhibitor, and diagram of the sampling and phenotyping timepoints. **(A)** Phenotyping and sample collection timepoints for RNA-Seq and LC-MS/MS, respectively. **(B)** Chilling stress phenotypes of 9311 and DC90 with exogenous application of ABA and its biosynthetic inhibitor Na_2_WO_4_. **(C)** Survival rate of 9311 and DC90 under chilling stress with/without exogenous ABA/Na_2_WO_4_ application. Scale bars = 5 cm in **(B)**. Three biological replicates were tested.

The growth of rice seedlings and chilling-stress phenotyping were performed according to previously described methods (Cen et al., 2018). The non-lethal 72-h/24-h chilling/recovery treatment regime was applied for ‘omics’ sample collection. Three and six biological replicates of whole plant samples were collected for RNA-Seq and LC-MS/MS, respectively. The samples for chilling stress phenotyping and measurements of physiological indexes were harvested based on two treatment regimes: one was the same as those for omics, and the other consisted of 120-h/12-h chilling/recovery to examine the ultimate changes of stress-induced biomarkers (Figure 1A and Supplementary Figure S1). For ABA application assay, four-leaf-stage seedlings were treated with or without 95.0 μM ABA or 10.0 mM sodium tungstate (Na_2_WO_4_) in nutrient solution under chilling stress for 5 days, then recovered at 28°C for 12 h and examined phenotypic changes (Matsuda et al., 2016). Three biological replicates were tested for the assay.

### Transcript Profiling

Sequencing libraries were generated using the NEBNext UltraTM RNA Library Prep Kit for Illumina (NEB, United States) following the manufacturer’s recommendations. PCR was performed with size-selected, adaptor-ligated cDNA using Phusion High-Fidelity DNA polymerase and the products were purified (AMPure XP system). The sequencing was performed on an Illumina 2500 platform, and paired-end reads were generated.

The clean reads were mapped to the reference genome (Rice Genome Annotation Project Release 7^1^) using Hisat2 tools. DEGs between each treated timepoint and untreated control were detected using DEseq with | log2(FC)| ≥ 2 and FDR ≤ 0.001 as thresholds to identify chilling- and recovery- induced DEGs, respectively. Raw data (raw reads) were deposited to gene expression omnibus (GEO) database of NCBI (accession number: GSE143878^2^).

### Metabolomic Profiling

Metabolites were extracted as described previously (Meng et al., 2019). The LC-MS/MS data were obtained using Nano LC 1000 LC-MS/MS with a Proxeon EASY-nLC 1000 coupled to a Thermo Fisher Q Exactive system. Metabolite identification was performed by automated comparison of their detected ion features with a reference library of chemical standard entries, including retention time, molecular weight (m/z), preferred adducts, in-source fragments and their associated MS/MS2 spectra. The raw area count for each metabolite was rescaled through the division of each sample value by the median value for this specific metabolite to obtain the scaled amount for better data visualization. DAMs between each treated timepoint and the untreated control was determined with the threshold of | log2(FC)| ≥ 1 and *P* ≤ 0.05, respectively.

### Data Integration and Statistical Analysis

SIMCA-P + 14.1 was employed to perform multivariate analysis and principal component analysis (PCA) and generate the scoring and loading plots. Transcriptomic and metabolomic data were integrated using O2PLS method which built in the SMICA-P + 14.1 (Umetrics, Umea, Sweden) to identify variables of joint variation between the two blocks (transcript block: X and metabolite block: Y) and that were unique to either block. The data were subjected to log10 transformation and Pareto (Par) scaling before modeling. Both datasets were then scaled to an equal total sum of squares to avoid the dominance of any dataset. A variables importance on the projection (VIP) score ≥ 1.1 and *P* ≤ 0.05 (Student’s *t*-test in the univariate analysis) were used as the threshold for identifying significant joint variation variables between two datasets (chilling-/recovery-induced transcript and metabolite dataset). Subsequently, an OPLS-DA analysis was performed based on the joint variation to discriminate the implicit class information between the classes (DC90: tolerant and 9311: sensitive classes) that were captured by related structures (Supplementary Figure S1) (Bylesjö et al., 2006). The chilling and recovery discriminatory variable set (subsets of DEGs and DAMs) were finally determined with the threshold of VIP ≥ 1.0, respectively.

Statistical analysis was perform using GraphPad 8.0 software. Multiple *t*-test was used to identify DEGs and DAMs (*P* ≤ 0.05) in the univariate analysis. The false discovery rate (FDR) was used to correct for multiple comparisons of all identified genes/metabolites. The R package ggcorrplot (v 0.1.3) was used to perform the Pearson’s coefficient correlation analysis.

### Quantitative Real Time Reverse Transcription PCR Analysis

Total RNA was isolated using TRIzol reagent following the manufacturer’s instructions. cDNA synthesis was performed by reverse transcription (RT) with the Thermo Scientific RevertAid First Strand cDNA Synthesis Kit (Cat# K1622) according to the manufacturer’s protocol. The primers for related genes were obtained from qPrimerDB^3^ (Lu et al., 2018). qPCR was performed using a Roche LightCycler 480 Real-Time PCR System in 10 μL reactions with the SYBR Green PCR Master Mix kit (Bio-Rad, United States) to detect the relative expression of these genes following the manufacturer’s protocol. The relative expression of each gene was calculated according to the 2^–△^^△^^CT^ method (Livak and Schmittgen, 2001). The *Actin* gene (LOC_Os11g06390) was used as an endogenous reference for qPCR.

### Targeted Quantification of Phytohormone

Phytohormone was extracted as described previously (Gong et al., 2016). The samples were subjected to UHPLC-QTRAP (AB SCIEX, United States). The flow rate was 400 μL/min, and the injection volume was 4 μL. Operation parameters of the ion source were as follows: MRM mode: source temperature 500°C, Ion Source Gas1 (Gas1): 45, Ion Source Gas2 (Gas2): 45, Curtain gas (CUR): 30, ion Spray Voltage Floating (ISVF)-4500 V.

### Generation of CRISPR/Cas9 Knockout Lines

CRISPR/Cas9 genome-targeting system was used to create the knockout lines. The plasmids were constructed as described previously (Ma et al., 2015). The resulting constructs with specific target sites were introduced into the *Japonica* rice ZH11 via *Agrobacterium tumefaciens-*mediated transformation. The homozygous knockout lines were selected for further phenotypic analysis.

### Relative Water Content (RWC) and Root Osmotic Exudation Measurements

The samples were collected and weighed to determine their fresh weight (FW), then oven-dried to get dry weight (DW). The RWC was calculated using the following equation:

$$\text{RWC}\left(\%\right)=\frac{\text{FW}-\text{DW}}{\text{FW}}\times 100\%$$

The osmotic exudation was expressed as the volume of exudation per hour per gram of root dry weight (g h^–1^g^–1^RDW) (Murai-Hatano et al., 2008). Rice plants were cut off at a distance of 2–3 cm from the base. The xylem bleeding saps were collected into a tube with a cotton ball for 12 h and weighed. The roots were collected and the RDW was measured.

### Analysis of the Stomatal Aperture Movement

The second leaves of chilling/recovery-treated seedlings were cut into 5-mm squares and immediately frozen in liquid nitrogen. The leaf stomatal apertures were examined under a FEI QRATRO S scanning electron microscope (Thermo Fisher Scientific, United States) with a freezing stage. The stomatal opening status was defined and analyzed (Matsuda et al., 2016).

### Examination of ROS and ROS Enzyme Activity

The second leaves of rice plants (0.15 g fresh weight) were ground in liquid nitrogen to a fine powder and then thoroughly resuspended in 1.35 mL phosphate buffered saline (PBS) buffer (10 mM, pH 7.2). After centrifugation, the supernatant was collected for subsequent assays. The contents of H_2_O_2_, catalase (CAT), peroxidase (POD), superoxide dismutase (SOD), and ascorbate peroxidase (APX) were measured using an enzyme-linked immunosorbent assay (ELISA) kit (Mskbio Company, Wuhan, China) following the manufacturer’s instructions. The amount of total proteins was quantified by comparison with a standard curve using BCA Kit (Thermo BCA protein assay kit). The accumulation of H_2_O_2_ in the leaves and in leaf guard cells were respectively estimated by DAB and 10 μM H_2_DCFDA staining as described previously (Cui et al., 2015; Wu et al., 2016).

## Results

### Exogenous Application of ABA Improves the Chilling Tolerance of 9311

Our previous studies have demonstrated a contrasting tolerance phenotype between 9311 and DC90 in response to chilling stress. Subsequently, we attempted to examine whether *CTS-12*-mediated chilling tolerance was related to ABA. Exogenous application of ABA showed that ABA significantly improved the chilling tolerance of 9311. Moreover, the treatment of chilling-treated seedlings with Na_2_WO_4_, an inhibitor of ABA biosynthesis, resulted in reduced chilling tolerance of DC90 (Figures 1B,C). These results suggested that ABA biosynthesis might be impaired in 9311 and results in a chilling sensitive phenotype.

Complex bioprocesses are involved in the responses of rice to chilling stress. Therefore, to further unveil the impact of *CTS-12* on ABA signaling-induced multilevel regulation, we investigated the transcriptomic and metabolomic responses of 9311 and DC90 under chilling and recovery treatment using RNA-Seq and LC-MS/MS. Two datasets were integrated by O2PLS and OPLS-DA modeling to identify valuable information that might provide clues for interpretation of chilling tolerant mechanisms.

### Integration and Correlation of Transcriptome and Metabolome

**FIGURE 2:**
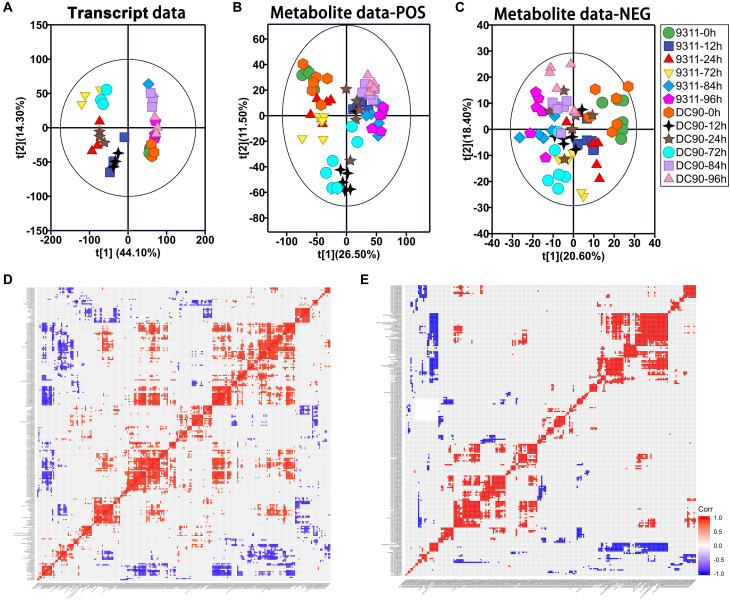
Principal component analysis (PCA) of the transcript and metabolite profiles of 9311 and DC90 samples and correlation analysis of discriminatory DEGs and DAMs, respectively. **(A)** PCA revealed transcriptomic shifts. **(B)** PCA revealed metabolomic shifts (POS, positive mode). **(C)** PCA revealed metabolomic shifts (NEG, negative mode). **(D)** The significant correlation among chilling-induced DEGs/DAMs. **(E)** The significant correlation among recovery-induced DEGs/DAMs. The t[1] represents the scoring values of the first principal component, and the t[2] represents the scoring values of the second principal component in **(A–C)**. Rectangles in **(D,E)** represent Pearson correlation values of pairs of DEGs or DAMs (see correlation color key). Colored rectangles represent a significant correlation (*P* ≤ 0.05).

Principal component analysis analysis was initially performed to detect the variations induced among the timepoint samples with the imposition of stress treatment (Figures 2A–C). In the transcript dataset, PC1 showed clearly separated between the chilling-treated timepoints and 0 h control/recovery timepoint samples. While clear separation among 12, 24, and 72 h of chilling stress treatments and among 0, 84, and 96 h of the control and recovery samples were observed in PC2, respectively. However, clear separation between the two genotypes was not observed in the transcript data PCA plot (Figure 2A), indicating stronger impacts of treatment time on the transcriptome than the genotype. In POS mode of LC-MS/MS, PCA plotting showed a clear separation between the recovery and chilling treatment of 9311 and DC90 with the exception of 12-h samples of 9311 in the PC1. PC2 also showed a separation among chilling timepoints and, interestingly, a shift of the 12- and 72-h samples of DC90 from the corresponding timepoints of 9311 (Figure 2B), showing the impact of *CTS-12* on the separation of the genotypes at metabolic level. In NEG mode, however, a clear separation was only observed between the control and treated samples (Figure 2C).

We integrated the RNA-Seq and LC-MS/MS datasets using O2PLS and OPLS-DA modeling sequentially (Supplementary Table S1). In the modeling of O2PLS, 1,230 variables (940 transcripts and 290 metabolites) were identified as joint variation between the two datasets with a threshold of VIP ≥ 1.1 and *P* ≤ 0.05 (Supplementary Table S2). Then, OPLS-DA analysis was performed to further screen the discriminatory variables from joint variation that can discriminate chilling tolerant and sensitive classes (DC90 and 9311) (Supplementary Tables S3, S4 and Supplementary Figures S3A–D). Permutation test was performed to check the validity and degree of overfit of the OPLS-DA model (Supplementary Figure S2). Finally, 231 DEGs/85 DAMs in the chilling treatment and 278 DEGs/39 DAMs in the recovery treatment were identified as discriminatory variables, respectively (Supplementary Table S5). Among these, both DEGs and DAMs showed period-specific patterns (Supplementary Figures S3E,F).

We then performed a Pearson’s correlation analysis among discriminatory DEGs and DAMs. During both treatment periods, significant positive and negative correlations were clearly observed among the transcripts-metabolites and themselves (Figures 2D,E). All the features shown here primarily indicated the relationships between metabolomic and transcriptomic data statistically.

### Metabolic Shift Under Chilling and Recovery Treatments

**FIGURE 3:**
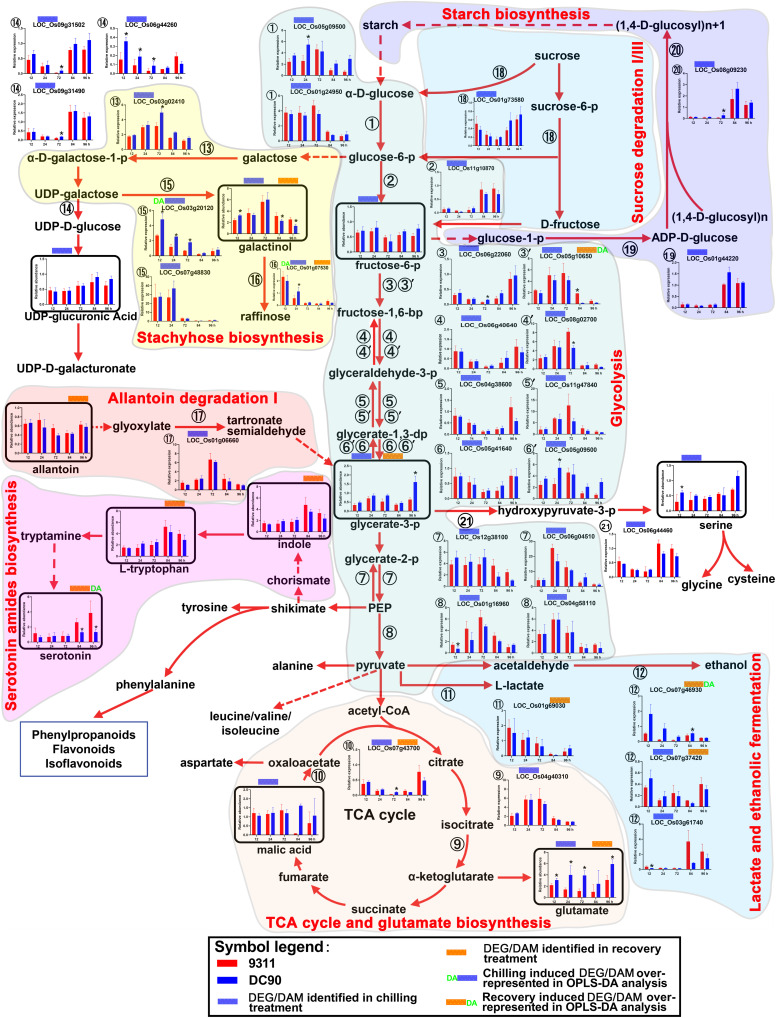
Chilling- and/or recovery-induced DEGs and DAMs mapped to carbohydrate metabolism and their related pathways. The boxed bar plots represent the metabolites with relative abundance alterations at certain timepoints in response to chilling (blue bar at the top) or recovery (orange bar at the top) treatment. The relative expression level alteration plots of genes encoding related enzymes are numbered accordingly for the space limitation. The blue and orange bars at the top of the plot represent the transcripts or metabolites that were significantly altered in response to chilling (blue bar) and/or recovery (orange bar) treatment. The plot of transcripts or metabolites with green letters DA to the left of the blue bar or to the right of the orange bar represent transcript or metabolite members of discriminatory DEGs/DAMs that play key roles in differentiating the chilling tolerance capacity of 9311 and DC90. The red and dashed arrows represent overall upstream/downstream relationships and the conversion catalyzed by multiple steps in the related pathways, respectively. *Significant at *P* ≤ 0.05 level according to Student’s *t* test.

**FIGURE 4:**
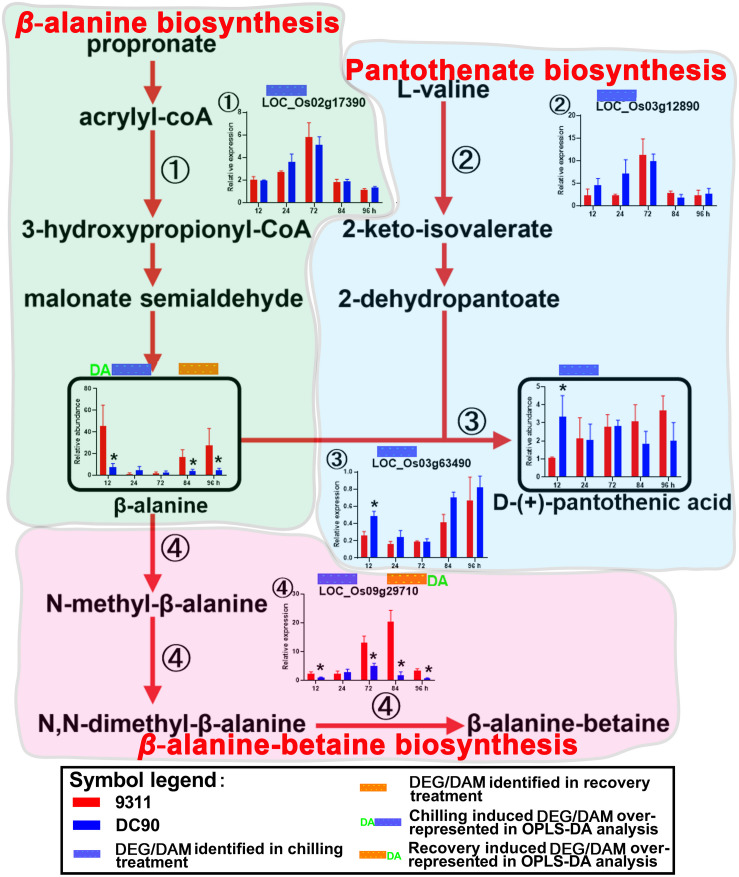
Chilling- and/or recovery-induced DEGs and DAMs mapped to β-alanine biosynthesis and its related pathways. The boxed bar plots represent the metabolites with relative abundance alterations at certain timepoints in response to chilling (blue bar at the top) or recovery (orange bar at the top) treatment. The genes encoding the related enzymes with relative expression level alteration plots are numbered accordingly. The blue and orange bars at the top of the plot represent the transcripts or metabolites that are significantly altered in response to chilling (blue bar) and/or recovery (orange bar) treatment. The plot of transcript or metabolites with the green letters DA to the left of the blue bar or to the right of the orange bar represents the transcript or metabolite members of discriminatory DEGs/DAMs that play key roles in differentiating the chilling tolerance capacity of 9311 and DC90. The red arrows represent overall upstream/downstream relationships in the related pathways, respectively. *Significant at *P* ≤ 0.05 level according to Student’s *t* test.

**FIGURE 5:**
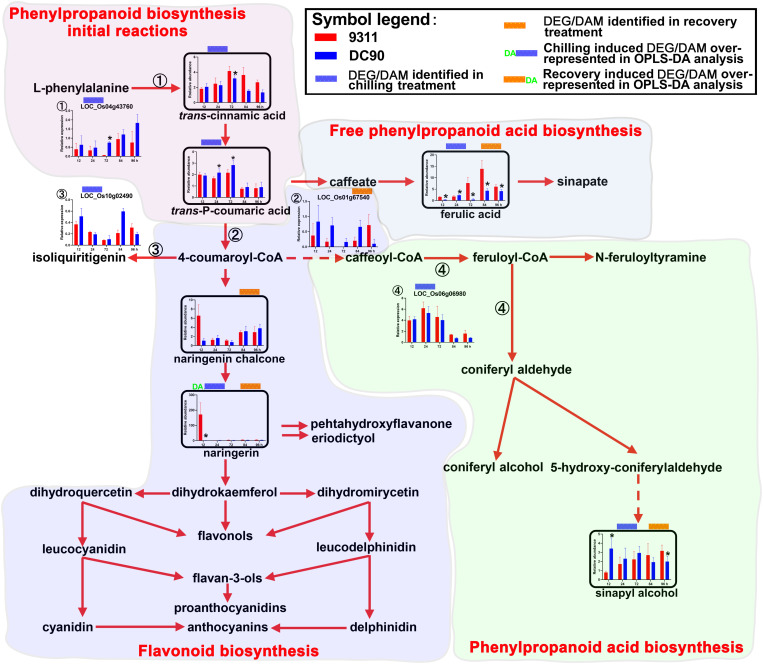
Chilling- and/or recovery-induced DEGs and DAMs mapped to phenylpropanoid/flavonoids biosynthesis pathways. The boxed bar plots represent the metabolites with relative abundance alterations at certain timepoints in response to chilling (blue bar at the top) or recovery (orange bar at the top) treatment. The genes encoding related enzymes with relative expression level alteration plots are numbered accordingly. The blue and orange bars at the top of the plot represent the transcript or metabolite that was significantly altered in response to chilling (blue bar) and/or recovery (orange bar) treatment. The plot of transcripts or metabolites with the green letters DA to the left of the blue bar or to the right of the orange bar represent the transcript or metabolite members of discriminatory DEGs/DAMs that play key roles in differentiating the chilling tolerance capacity of 9311 and DC90. The red and dashed arrows represent overall upstream/downstream relationships and the conversion catalyzed by multiple steps in the related pathways, respectively. *Significant at *P* ≤ 0.05 level according to Student’s *t* test.

**FIGURE 6:**
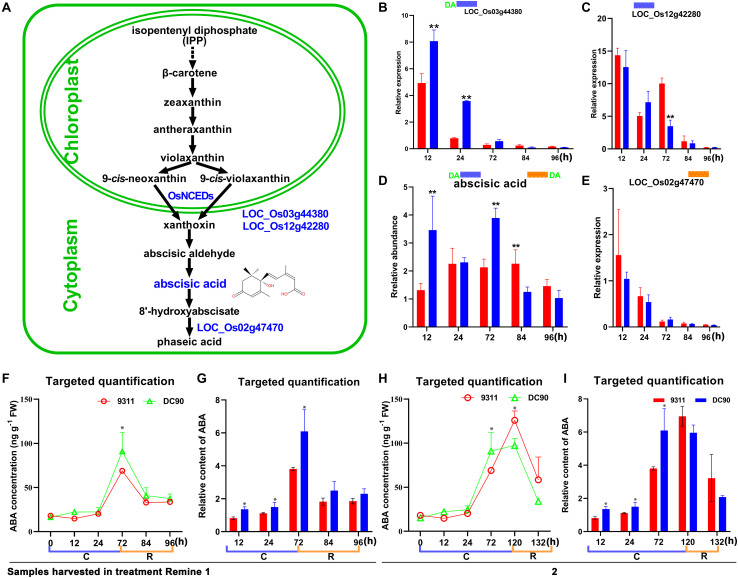
Chilling- and/or recovery-induced DEGs and DAMs mapped to the abscisic acid metabolic pathway. **(A)** Abscisic acid metabolism pathway and mapped DEGs and DAMs. **(B,C)** Changes in *LOC_Os03g44380* and *LOC_Os12g42280* in 9311 and DC90, respectively. **(D)** Changes in abscisic acid in 9311 and DC90. **(E)** Changes in *LOC_Os02g47470* in 9311 and DC90. **(F,G)** The concentrations and relative contents of ABA determined by targeted quantification under chilling and recovery treatment (0–96 h, Regime 1 in Figure 1A), respectively. **(H,I)** The concentrations and relative contents of ABA determined by targeted quantification under chilling and recovery treatment (0–132 h, Regime 2 in Figure 1A), respectively. The bar plots in **(B–E)** represent the metabolites with relative abundance alterations at certain timepoints in response to chilling (blue bar at the top) or recovery (orange bar at the top) treatment. The plot of transcripts or metabolites with the green letters DA to the left of the blue bar or to the right of the orange bar represent transcript or metabolite members of discriminatory DEGs/DAMs that play key role in differentiating the chilling tolerance capacity of 9311 and DC90. The black and dashed arrows in **(A)** represent overall upstream/downstream relationships and conversion catalyzed by multiple steps in the related pathways, respectively. *, ** in **(B–I)**. Significant at *P* ≤ 0.05 and 0.01 levels according to Student’s *t* test, respectively.

Pathway integration based on common and discriminatory DEGs/DAMs was performed using RiceCyc^4^ (Supplementary Tables S5-1, 2, 3, 4). In the chilling period, a total of 77 DEGs and 28 DAMs were mapped to different steps of 58 metabolic pathways. Twenty one DEGs and sixteen DAMs were mapped to 28 pathways under recovery (Supplementary Tables S5-9, S5-10). Not all but those might relate to abiotic/biotic stress responses were addressed herein. These pathways included carbohydrate metabolism, amino acid metabolism, ABA biosynthesis, photosynthesis/photorespiration, and phenylpropanoid/flavonoid biosynthesis, among others (Figures 3–6 and Supplementary Figures S4–S7).

### The Perturbation of Carbohydrate Metabolism

Carbohydrate metabolic pathways, especially glycolysis and the tricarboxylic acid (TCA) cycle, are the central pathways of metabolism. Regarding the chilling induced responses, glycolysis was perturbed in both genotypes, which was manifested by the mildly decreased content of fructose-6-phosphate (F6P) and glycerate-3-phosphate (G3P) in 12-h of chilling treatment and the down-regulation of enzymes that catalyzes the reactions of several steps of the pathway (Figure 3). Four genes, which encode the enzymes for glycolysis, were down-regulated at all three timepoints of chilling treatment (➂➃➄➅), although another set of genes encoding the isoenzymes for this pathway (➂′➃′➄′➅′) exhibited up-regulated expression. The discrepancy of expression patterns here might result from the subcellular compartmentalization of the enzymes in the cells (Figure 3). In particular, *LOC_Os06g22060* encoding F6P kinase, which catalyzes the phosphorylation of F6P to produce fructose-1,6-bisphosphate, is a rate-limiting step of glycolysis. For the case of G3P, however, its content was almost recovered to untreated control levels in the next two timepoints of chilling treatment, potentially due to the up-regulation of transcript *LOC_Os01g06660* in the allantoin degradation pathway (Figure 3). Correspondingly, transcriptional up-regulation of enolases (*LOC_Os12g38100*/*LOC_Os06g04510*) and pyruvate kinase (*LOC_Os01g16960*/*LOC_Os04g58110*) increased the intermediate supplies of glycolysis for the TCA cycle (Figure 3), which was reflected by the mildly increasing content of malic acid and expression of a key enzyme, isocitrate dehydrogenase (*LOC_Os04g40310*), and by the accumulation of glutamate. For F6P, the decreases in content might result from the activation of stachyose biosynthesis and inhibition of carbon fixation during photosynthesis and photorespiration (Figure 3 and Supplementary Figures S4A,B). Particularly, galactinol and raffinose are the products of the stachyose biosynthesis pathway, which branch off from the glycolysis intermediate glucose-6-phosphate. Intriguingly, the content of galactinol accumulated during chilling treatment and showed a significant difference between 9311 and DC90 at the 12-h timepoint. In line with this tendency, four genes, *LOC_Os03g02410*, *LOC_Os03g20120*, *LOC_Os07g48830*, and *LOC_Os01g07530*, which encode enzymes for three steps of this pathway, showed an elevated expression, and the genes encoding galactose degradation enzyme were down-regulated (1⃝4 in Figure 3). Among these, both *LOC_Os03g20120* and *LOC_Os01g07530* were detected as discriminatory DEGs in OPLS-DA modeling, indicating the important role of this pathway in differentiating the chilling tolerance of 9311 and DC90. *LOC_Os03g20120*, a gene encoding glycosyl transferase 8, catalyzes the transfer of glycosyl from UDP-galactose to myoinositol to produce galactinol, and glycosyltransferase (encoded by *LOC_Os01g07530*) further transfers galactosyl to sucrose to produce raffinose, although it was undetectable in our study (Figure 3). Moreover, the up-regulation of *LOC_Os05g09500* and *LOC_Os01g24950*, two genes encoding hexokinase for catalysis of glucose phosphorylation in the first step of glycolysis, and the down-regulation of *LOC_01g44220*, *LOC_Os08g09230*, and *LOC_Os01g73580*, three DEGs encoding glucose-1-phopshate adenyltransferase, starch synthase III, and glycosyl hydrolases for starch biosynthesis (*LOC_01g44220*/*LOC_Os08g09230*) and sucrose degradation (*LOC_Os01g73580*), suggested the increases in starch degradation accompanying the inhibition of starch biosynthesis under chilling stress. During the recovery period, the reduced abundance of G3P was notably recovered in DC90 but not 9311 after 96-h of recovery growth, suggesting the recovery of glycolysis and photosynthesis in the chilling-tolerant genotype. Altogether, carbohydrate metabolism in rice plants was re-programmed to maintain cellular homeostasis in response to chilling stress.

### The Dynamic Changes in Amino Acid Metabolism

The accumulation of amino acids in response to environmental stress has been reported in many plant species (Fabregas and Fernie, 2019). In agreement with these previous studies, amino acids and their derivatives, including L-tryptophan, L-tyrosine, β-alanine, glutamate, L-arginine, methionine, L-isoleucine, and serotonin, were found significantly accumulated with the exception of serine in present study (Figures 3, 4 and Supplementary Figures S5–S7). L-tyrosine, methionine, and L-isoleucine showed enhanced abundance during chilling stress (Supplementary Figures S6, S7), whereas tryptophan, L-arginine, and serotonin were accumulated in 9311 and DC90 during recovery (Figure 3 and Supplementary Figure S5). Furthermore, β-alanine and glutamate were accumulated in both genotypes in two treatment stages (Figure 4 and Supplementary Figure S5). Particularly, β-alanine was detected as a discriminatory DAM in chilling stress treatment and exhibited greater fluctuation in 9311 throughout the treatment period, while in DC90 it maintained a relatively stable level (Figure 4). The increasing accumulation of β-alanine might supply abundant substrate for β-alanine-betaine biosynthesis. *LOC_Os09g29710*, a discriminatory DEG detected during recovery, encodes an enzyme involved in three reaction steps of the pathway (Figure 4); similar to the pattern of β-alanine during recovery treatment, it also had a greater abundance in 9311 and a stable level in DC90. β-alanine also participates in pantothenic acid biosynthesis; however, pantothenic acid was significantly accumulated in DC90 after 12-h of chilling stress.

Glutamate links carbohydrate and amino acid metabolism via the TCA cycle. α-ketoglutarate, as an important intermediate of the TCA cycle, is converted to glutamate by the reaction catalyzed by aminotransferase. As mentioned above, the samples of 9311 and DC90 accumulated glutamate in both treatment periods with significantly different magnitudes. DC90 exhibited a greater accumulation at almost all timepoints of the treatments (Supplementary Figure S5). Proline acts as a well-known osmolyte during the plant stress response. However, proline was undetectable in the present study. Nonetheless, in contrast to 9311, *LOC_Os10g40360*, which encodes proline oxidase for the degradation of proline and exhibited a greater up-regulation at 24 h treatment in DC90, might be one explanation for the higher accumulation of glutamate during chilling stress. Moreover, glutathione, an important antioxidant in plants, appeared to accumulate after the imposition of chilling (Supplementary Figure S5). These results suggested that the flux of glutamate was mainly directed toward the biosynthesis of glutathione. Moreover, in recovery period, two genes, *LOC_Os01g40870* and *LOC_Os04g56400*, were identified as discriminatory DEGs; *LOC_Os01g40870*, which encodes aldehyde dehydrogenase for catalyzing the conversion of L-glutamate-5-phosphate to L-glutamate-semialdehyde, was significantly inhibited in DC90, whereas it was up-regulated in 9311. *LOC_Os04g56400*, encoding glutamine synthetase for glutamine biosynthesis, was significantly down-regulated in DC90 in contrast to 9311 (Supplementary Figure S5). The dynamic changes in the two genes might also result in the differential increased accumulation of glutamate in both genotypes during recovery growth.

L-methionine is one of the substrates for ethylene biosynthesis. The accumulation of methionine under chilling stress might supply sufficient substrate for this process. Although we were unable to detect ethylene in our experimental scheme, the targeted quantification of the precursor of ethylene, ACC, which displayed strong differential accumulation in 9311 and DC90 at 72 h, and the corresponding upregulation of the gene *LOC_Os02g53180*, encoding an enzyme required for the conversion of 3-aminocyclopropane-1-carboxylate (ACC) to ethylene, indirectly supported the differential accumulation of ethylene in two genotypes (Supplementary Figure S6).

Serotonin is produced by the decarboxylation of tryptophan and the reduction of tryptamine (Figure 3). Serotonin accumulation has been reported in plant leaves in response to abiotic and biotic stress (Dharmawardhana et al., 2013; Gupta and De, 2017). In contrast to DC90, significant accumulation of serotonin was observed in 9311 during recovery (Figure 3). Serotonin was also a discriminatory DAM, suggesting that the over-accumulation of serotonin might cause injury to 9311 in the recovery stage.

### The Up-Regulation of Phenylpropanoid/Flavonoid Biosynthesis

Secondary metabolites such as phenylpropanoids, flavonoids, and their derivatives are synthesized from phenylalanine via a core pathway that is conserved in plants (Tohge et al., 2013). They are important groups of compounds that are essential for plant survival in response to environmental stresses (Fraser and Chapple, 2011). Notably, five phenylpropanoids, *trans*-cinnamic acid, *trans*-*P*-coumaric acid, ferulic acid, sinapyl alcohol, and naringenin chalcone, and one flavonoid (naringenin), were consistently accumulated in 9311 and DC90 under the induction of chilling and recovery treatments, despite the opposite regulatory pattern of the DEGs *LOC_Os04g43760*, *LOC_Os10g02490*, and *LOC_Os01g67540* (Figure 5). It is worth noting that naringenin was identified as a discriminatory DAM during chilling stress with strong accumulation in 9311 in contrast to DC90 at 12 h, suggesting its key roles in chilling tolerance variation between two genotypes. Moreover, a significant difference in the abundance of other phenylpropanoids was detected between 9311 and DC90 at certain timepoints, also suggesting their possible roles in the chilling tolerance variation (Figure 5).

### The Differential Accumulation of ABA

**FIGURE 7:**
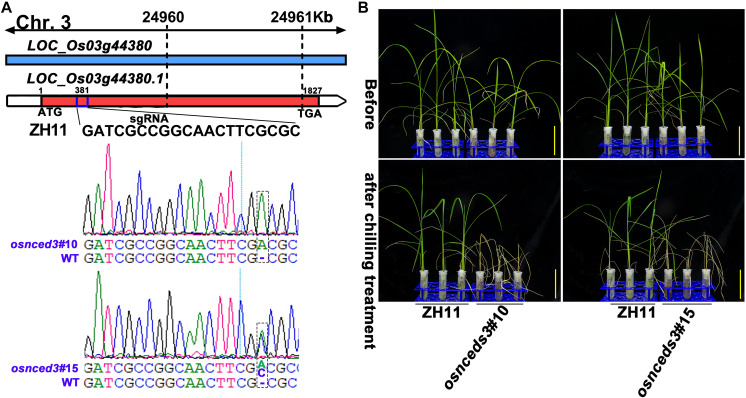
CRISPR/Cas9-edited *OsNCED3* gene and the chilling stress phenotype of CRISPR/Cas9-edited lines. **(A)** Targeted site designed in the coding sequence of *OsNCED3* and the sequences of target site in two knockout lines. **(B)** The chilling stress phenotype of *OsNCED3* CRISPR/Cas9-edited knockout lines. Scar bar = 5 cm.

Chilling stress induced the accumulation of endogenous ABA in plants (Mahajan and Tuteja, 2005; Chawade et al., 2013; Sah et al., 2016). The enhancement of chilling tolerance of 9311 upon exogenous application of ABA suggested that *CTS-12* might regulate the chilling stress response in an ABA-dependent manner (Figures 1B,C). To further confirm this relationship, we paid more attention to the changes in chilling-induced ABA level. In line with previous descriptions, ABA was accumulated in both genotypes and exhibited significantly higher accumulation in DC90 after 12- and 72-h of chilling stress in contrast to 9311 (Figure 6D). Conversely, higher accumulation of ABA was observed in 9311 at 84-h timepoint of recovery. The enhanced level of ABA suggested the activation of ABA biosynthesis or down-regulated catabolism in response to chilling stress in rice plants. Correspondingly, the expression level of DEG *LOC_Os03g44380* (*OsNCED3*), which encodes a key enzyme 9-*cis*-epoxycarotenoid dioxygenase (NCED) for ABA biosynthesis, was increased with greater magnitude in DC90 after 12- and 24-h of chilling stress treatment (Figures 6A,B). Additionally, another DEG, *LOC_Os12g42280* (*OsNCED5*), encoding an isoenzyme, was also up-regulated during chilling treatment (Figure 6C). During recovery treatment, the expression of *LOC_Os02g47470* (*OsABA8ox1*), a gene annotated as cytochrome P450 with oxidoreductase activity in the downstream of ABA catabolism, was repressed to a greater extent in both genotypes (Figure 6E), which might help to retain a certain level of ABA in response to stresses triggered by the dramatic temperature change at the onset of recovery stage, especially in 9311 (Figure 6D). However, a high level of ABA in 9311 implied that it was experiencing more higher level of stresses and injuries, which would inhibit post-chilling stress growth. Intriguingly, *OsNCED3* and ABA were simultaneously detected as discriminatory factors, suggesting the involvement of ABA in *CTS-12* mediated chilling stress regulation. Therefore, to further validate this idea, phytohormones were quantified using targeted LC-MS/MS (Supplementary Figure S8) and *OsNCED3* was undergone CRISPR/Cas9 edited to confirm whether it is correlated with chilling stress response (Figures 7A,B). Consistently, the content of ABA was increased in both genotypes in response to chilling treatment and showed significant a higher level in DC90 than 9311 at 72 h in both treatment regimes (Figures 1A, 6F,H). Conversely, however, the content of ABA was higher in 9311 than DC90 after 120-h of chilling in the Regime 2 (Figures 1A, 6H). Furthermore, the relative alteration pattern of ABA levels calculated based on targeted quantification data was in line with those data acquired in the non-targeted method (Figures 6D,G,I). Strikingly, the knockout of *LOC_Os03g44380* gene in *japonica* rice ZH11 significantly impaired its chilling tolerance (Figure 7).

Moreover, the shifts of key discriminatory DEGs identified in this study were validated by qRT-PCR. The relative expressions of all the tested DEGs exhibited similar alteration patterns with significant differences in the certain timepoints between DC90 and 9311 under chilling and recovery stress treatment in contrast to RNA-seq, indicating the reliability of RNA-seq data (Supplementary Figure S9 and Supplementary Table S6).

### The Differences in Stomatal Opening and Water Loss in 9311 and DC90 Under Chilling and Recovery Treatments

**FIGURE 8:**
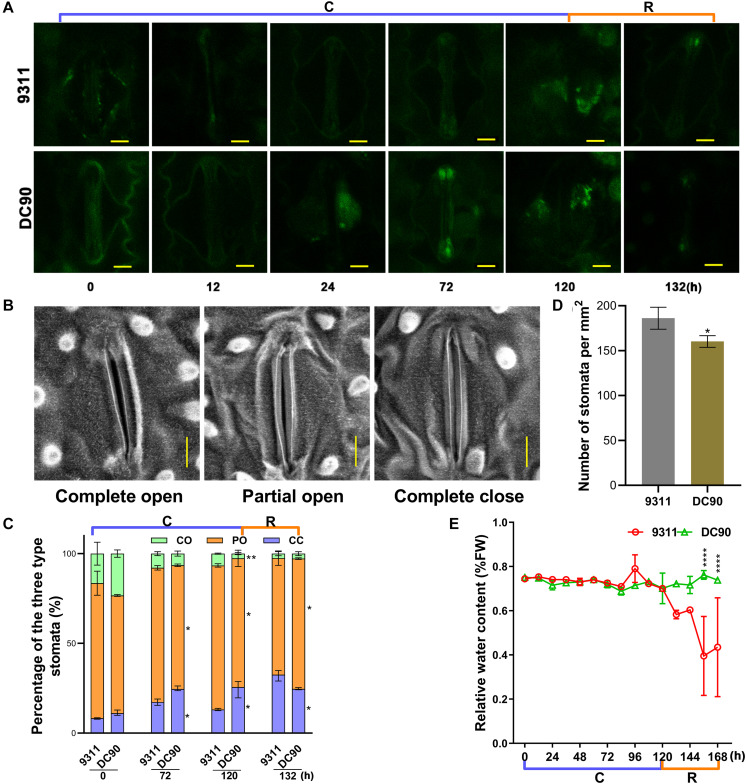
The accumulation of H_2_O_2_ in stomatal guardcells and stomatal movements of leaves in response to chilling stress and recovery treatment. **(A)** H_2_O_2_ accumulation in leaf guard cells of 9311 and DC90 at chilling and recovery treatment timepoints. **(B)** Leaf stomatal opening during chilling and recovery treatment. **(C)** The percentages of three levels of stomatal opening in 9311 and DC90 (*n* = 228 stomata for 9311; *n* = 208 stomata for DC90). **(D)** Stomatal density of the middle part of leaves of 9311 and DC90 (*n* = 8). Four random fields were used in each repeat. **(E)** Relative water content of 9311 and DC90 (*n* = 3). Scale bar, 5 μm in **(A,B)**; data are presented as the mean ± SD in **(C–E)**. * Significant at *P* ≤ 0.05 and **, ***, **** significant at *P* ≤ 0.01 according to Student’s *t*-test.

Chilling stress induces ROS accumulation in plant cells (Pareek et al., 2017). Although over-accumulation of ROS causes injury to plant cells, it is also considered as an important second messengers involved in the ABA-mediated stress-responsive signaling pathways, for example, guard cell stomatal movement (Agurla et al., 2018). Examination of ROS and the activities of scavenging enzymes showed that the contents of H_2_O_2_, SOA (total super oxidative anion), and the contents of their scavenging enzymes, for example CAT, POD, APX, and SOD, did not exhibit enhanced accumulation in 9311 and DC90 throughout the chilling and recovery treatment period, even when the chilling treatment time was extended to 120 h. More interestingly, all the indexes exhibited highly dynamic changes during the treatments; particularly, their levels were generally decreased at the first two timepoints (12- and 24-h) and recovered at the later timepoints (Supplementary Figures S10A–F,I–N). Moreover, the accumulation of any indicators at any treatment timepoints did not exceed those in the control samples. These indicating that the normal redox homeostasis was not disrupted in rice cells of either 9311 or DC90. Therefore, we speculated that chilling-induced injuries in rice were not associated with oxidative stress in the timeframe of our study. This notion was further strengthened by examining ROS, MDA, and relative electrolyte leakage levels of leaf samples in two chilling treatment regimes (Supplementary Figures S10G,H,O,P, S11A,B). However, microscopic analysis of H_2_DCFDA-stained leaves revealed a strong accumulation of H_2_O_2_ in stomatal guard cells in 24-, 72-, and 120-h chilling-treated samples of DC90, whereas a low level of H_2_O_2_ was observed in 9311 (Figure 8A), suggesting that H_2_O_2_ was only accumulated to a sub-injury level in response to chilling treatment. Therefore, scanning electron microscopy was used to examine the stomata status of chilling-treated leaf samples. By comparison with the untreated control, the percentages of completely closed stomata in the chilling- and recovery-treated samples ( 72-, 120-, and 132-h) were significantly elevated, whereas those of partially open stomata were lower in DC90 than 9311 (Figures 8B–D). The higher proportion of partially open and lower proportion of completely closed of 9311 leaf stomata resulted in higher water loss from stomata than root water uptake at the onset of the recovery stage, thereby leading to a chilling sensitive phenotype of rice (Figures 8C–E and Supplementary Figures S11C–F). Therefore, the impact of *CTS-12* on chilling tolerance phenotype was associated with the prevention of the respiration water loss through ABA-dependent stomatal regulation to maintain the balance between water uptake and water loss. The balance is prone to be impaired by environmental stresses.

## Discussion

In this study, to unveil the impact of *CTS-12* on ABA-dependent multilevel regulation, we integrated the transcriptomic and metabolomic data of chilling-treated 9311 and DC90 samples by O2PLS and OPLS-DA modeling. Our results revealed the involvement of ABA in *CTS-12*-mediated chilling tolerance variation (Figure 1). Additionally, carbohydrate-related metabolism, amino acid metabolism, and phenylpropanoid biosynthesis were significantly shifted and exhibited different magnitudes of alteration or opposite regulatory patterns between 9311 and DC90 as well.

### Impact of CTS-12 on the Differential Alterations of the Metabolism Pathways Might Contribute to Chilling Stress Tolerance

#### Carbohydrate Metabolism

Glycolysis and the TCA cycle have been previously reported to be inhibited under water stress (Yoshida et al., 2019). In line with these findings, the present study revealed altered glycolysis in 9311 and DC90 under chilling stress, as reflected by the decreasing content of F6P and the down-regulation of genes encoding enzymes involved in glycolysis (Figure 3). These might mainly result from the activation of stachyose biosynthesis, as manifested by the differential transcriptional up-regulation of the discriminatory genes *LOC_Os03g20120* and *LOC_Os01g07530* (higher levels in DC90) (Figure 3). Galactinol and raffinose are accumulated in response to various environmental stresses and serve as osmotic protectants, cell membrane stabilizers, and ROS scavengers (Nishizawa et al., 2008). Moreover, the up-regulation of enolases (*LOC_Os12g38100*/*LOC_Os06g04510*) and pyruvate kinase (*LOC_Os01g16960*/*LOC_Os04g58110*) might supply more G3P for the TCA cycle (Figure 3). Correspondingly, the levels of the TCA cycle intermediate, malic acid, were slightly increased with the concomitant down-regulation of *LOC_Os07g43700*, a gene encoding malic acid dehydrogenase for the oxidation of malic acid to oxaloacetate, and with the up-regulation of *LOC_Os04g40310*, which might indirectly lead to the strong accumulation of glutamate, especially the elevated levels observed in DC90 (Figure 3). Therefore, this scenario suggested a global picture of the reprogramming of carbohydrate metabolism in response to chilling stress in rice, that is, the glycolysis and TCA cycle energy production pathways were perturbed and redirected the metabolic flux branching off to stachyose biosynthesis, enhancing production of antioxidant galactinol, and to glutamate, a link between the TCA cycle and nitrogen metabolism. The higher activities of these pathways in DC90 might be associated with the improvement of chilling stress tolerance.

#### Amino Acid Metabolism

The accumulation of amino acids and their derivatives, including aromatic amino acids (AAAs: tryptophan, tyrosine, and phenylalanine) and branched-chain amino acids (BCAAs: leucine, isoleucine, and valine), in response to abiotic stresses have been extensively reported in recent literature (Obata et al., 2015; Todaka et al., 2017; Yoshida et al., 2019). The accumulation of AAAs expedites the production of secondary metabolites, which serve as endogenous antioxidants (Jia et al., 2016). BCAAs act as an alternative electron donor for the mitochondrial electron chain and alternative substrates for respiration under stress conditions (Pires et al., 2016). In agreement with those findings, our analysis revealed significant accumulations of L-tryptophan, L-tyrosine, β-alanine, glutamate, L-arginine, methionine, L-isoleucine, and serotonin in identified DAMs (Figures 3, 4 and Supplementary Figures S5–S7). β-alanine and glutamate were accumulated in the two genotypes in both stages (Figure 4 and Supplementary Figure S5).

β-alanine was regarded as a metabolite with a pivotal role in differentiating the chilling tolerance of 9311 and DC90. Based on the overview of β-alanine biosynthesis and its related pathways, the increased accumulation of β-alanine in 9311 might direct the metabolic flux toward β-alanine-betaine biosynthesis. However, the low level of β-alanine in DC90 suggested that the metabolic flux was redirected toward pantothenic acid biosynthesis (Figure 4); recent evidence has shown that pantothenic acid plays a central role in metabolism and in the resistance to biotic stress in soybean (Aliferis et al., 2014). Regarding the behavior of β-alanine under chilling stress, it was considered as a negative biomarker for chilling stress tolerance.

Glutamate serves as a primary precursor for stress-related metabolites, such as *c*-aminobutyrate and polyamines, via the formation of arginine (Mahdavi et al., 2015). Glutamate exhibited higher accumulation in DC90 in contrast to 9311 during the recovery (Supplementary Figure S5). In line with this tendency, glutathione appeared to accumulate during chilling stress, suggesting that the flux of glutamate was mainly directed toward the biosynthesis of glutathione. Moreover, the accumulation of methionine under chilling stress could be connected to the biosynthesis of ethylene, although ethylene could not be detected directly with the method used in this study (Supplementary Figure S6).

Therefore, we argue that the accumulation of amino acids in rice plants might play certain roles in the improvement of chilling stress tolerance, although it has been reported that the increased amino acid levels may not be a direct adaptive response to salt stress, but an indicator of general stress and cell damage (Urano et al., 2010).

#### Phenylpropanoid Biosynthesis

Secondary metabolites such as phenylpropanoids, flavonoids, and their derivatives are important groups of compounds with various physiological roles, including ROS scavenging, enzyme activation, photoprotection, and signal regulation, which are essential for plant survival in response to environmental stresses (Fraser and Chapple, 2011). Five phenylpropanoids, including *trans*-cinnamic acid, *trans*-P-coumaric acid, ferulic acid, sinapyl alcohol, naringenin chalcone, and one flavonoid naringenin, were consistently accumulated in 9311 and DC90 under chilling and recovery stresses (Figure 5). In particular, naringenin was identified as a discriminatory metabolite during chilling stress, which exhibited strong accumulation in 9311 in contrast to DC90 at 12 h, implying its effect on the variation in chilling tolerance between two genotypes. It has recently been shown that flavonols participate in the prevention of H_2_O_2_ generation and quench H_2_O_2_ once they are formed. The enhancement of flavonol biosynthesis is concomitant with the inactivation of antioxidant enzymes (Agati et al., 2011). Consistently, in our study, chilling stress tolerance of rice was, on the one hand, negatively correlated with the abundance of phenylpropanoids and flavonoids, and, on the other hand, positively correlated with the accumulation of H_2_O_2_ in stomatal guard cells.

### CTS-12 Mediated ABA-Dependent the Maintenance of Water Uptake and Loss Balance Confers Chilling Stress Tolerance in Rice

ABA is a key regulator in plant stress responses and regulates a series of physiological processes, including tolerance to abiotic stresses and stomatal closure (Fujii et al., 2009; Ma et al., 2009; Nishimura et al., 2009; Park et al., 2009; Chawade et al., 2013; Sah et al., 2016). In the integration analysis, the level of ABA was accumulated in both genotypes during chilling stress and positively correlated with the chilling tolerance phenotype of rice; for example, a higher level of ABA was accumulated in chilling-tolerant DC90 (Figure 6). Together with the trials of exogenous application ABA, the results suggested the pivotal role of ABA in *CTS-12*-mediated mechanisms that underlie chilling tolerance in wild rice.

When plants are exposed to abiotic stresses, including chilling, increased levels of ABA activate the guard cell stomata regulatory pathway (Nishimura et al., 2009; Umezawa et al., 2010). Chilling stress causes an imbalance of the water status in plants and induces the biosynthesis or redistribution of ABA; and ABA further induces the increases in the level of ROS (mainly H_2_O_2_) that promote stomatal closure to limit transpirational water loss in guard cells (Bright et al., 2006; Agurla et al., 2018). In agreement with these findings, the increased accumulation of ABA in DC90 (Figure 6) and the sublethal generation of H_2_O_2_ in leaf guard cells of DC90 (Figure 8) suggested the involvement of these metabolites in the regulation of stomatal opening under chilling stress. This suggestion was confirmed by examinations of stomatal opening, stomatal guard cell density, and leaf relative water content of chilling-treated rice plants (Figure 8). However, in our study, measurements of ROS and ROS scavenging enzymatic activity showed no stress-induced oxidative burst during chilling stress, even with an extension of the chilling treatment time to 5 days (Supplementary Figures S10, S11A,B). Therefore, together with these findings, we speculated that the chilling-triggered injuries in rice were unrelated to ROS directly but caused by the imbalance between water uptake and transpiration of rice plants due to low level of ABA accumulation and low level of stomatal closure under chilling stress. In the chilling-tolerant genotype DC90, *CTS-12*-mediated ABA accumulation coordinately regulated stomatal closure of guard cells to maintain the balance of water uptake and loss under chilling and recovery stresses.

In addition, several metabolites, including ABA, ethylene, flavonoids, ROS, and carbohydrates, were identified serving as key factors in chilling tolerance regulation in our results. This arouse us to question whether these factors participated in and how they coordinately functioned in the regulation of stomatal closure. However, we were unable to address these due to the resolution of our experimental scheme. The relationships among these factors in response to chilling stress in rice remain elusive and, thus, require further evaluation in future studies.

## Conclusion

**FIGURE 9:**
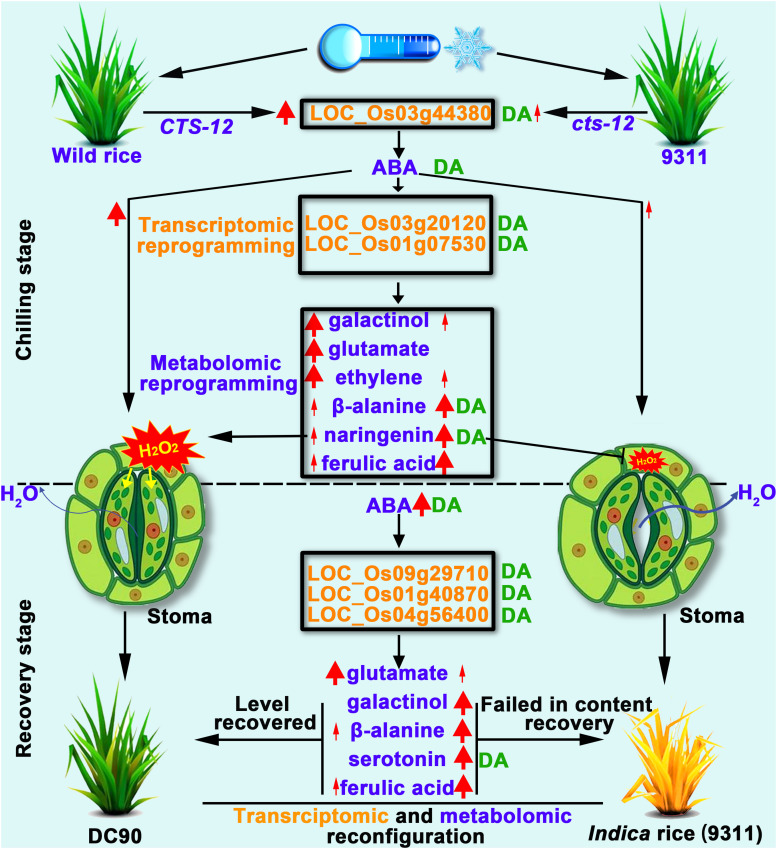
Working model proposed to depict the impact of *CTS-12* on the reprogramming of transcriptomic and metabolomic profiles in response to chilling stress. Thin and thick red arrows represent weak and strong inductions in response to chilling stress, respectively. Thin and thick dark blue arrows indicate lower and higher water loss from stomata under chilling stress, respectively. The green letters DA to the right of transcripts or metabolites represent discriminatory DEGs or DAMs identified by OPLS-DA modeling.

In this study, the integration of transcriptomic and metabolomic dataset were performed by O2PLS and OPLS-DA modeling to investigate the multilevel regulation of rice in response to chilling stress. Distinct transcriptomic and metabolomic responses were observed in 9311 and DC90 during the chilling and recovery periods. Therefore, we propose that, under chilling stress, the *CTS12*-mediated accumulation of ABA induced the signaling pathways that leads to the transcriptional alterations of chilling-responsive genes, and, in turn, triggers the metabolic shifts, including the over-representation of primary and secondary metabolic pathways, to coordinately regulate the stomatal movement of guard cells. *CTS-12* exert its effect on the differential regulation of stomatal closure, thereby resulting in the different water balance status in both genotypes during and after chilling stress. Furthermore, numerous metabolites, including galactinol, serotonin, β-alanine, naringenin, and sinapyl alcohol, recovered their levels from the stress-responsive state back to the regrowth stage (the levels comparable to their untreated controls) in recovery stage (Figure 9). These important processes occurred in the chilling-tolerant genotype (DC90) but not in the sensitive one (9311), thereby leading to a tolerant phenotype of rice under chilling stress.

## Data Availability Statement

The datasets presented in this study can be found in online repositories. The names of the repository/repositories and accession number(s) can be found in the article/ Supplementary Material.

## Author Contributions

JL, WC, and RL conceived and designed the experiments. WC, WZ, SL, MM, YC, ZZ, and HW performed all the lab experiments. WC, WZ, SL, MM, and JBL conducted the field trials and collected the samples for RNA-Seq and LC-MS/MS. JL and WC performed the data processing. JL drafted the manuscript. JL, RL, WC, WZ, SW, and MM revised the manuscript. All the authors approved the final version of the manuscript.

## Conflict of Interest

The authors declare that the research was conducted in the absence of any commercial or financial relationships that could be construed as a potential conflict of interest.

## Footnotes

1 http://rice.plantbiology.msu.edu/

2 https://www.ncbi.nlm.nih.gov/geo/query/acc.cgi?acc=GSE143878

3 https://biodb.swu.edu.cn/qprimerdb/

4 http://pathway.gramene.org/gramene/ricecyc.shtml
