# Supplementary material for: The Wild Rice Locus CTS-12 Mediates ABA-Dependent Stomatal Opening Modulation to Limit Water Loss Under Severe Chilling Stress
Source: Front Plant Sci. 2020 Oct 30;11:575699. doi: 10.3389/fpls.2020.575699 (PMC7661758; doi:10.3389/fpls.2020.575699)
Supplement: Supplementary file 6 [file Table_6.DOCX]

| **Table S6 The primer sequences of genes using for qPCR** | | |
| --- | --- | --- |
| **Gene ID** | **Forward Primers (5'-3') Reverse Primers (5'-3')** | |
|  |  |  |
| *LOC_Os03g44380* | CCAGGATATGCTCACATACAGC | GGAGAATCTCACCGAATTGGA |
| *LOC_Os03g20120* | TGCATCCCGTATCTTGGTATTT | CAAGCCTCGTTACATTTGTGAA |
| *LOC_Os01g07530* | AGGATGCGGAGTTCAAGTAC | TTGTCTTAGTCTCTTTCCAGCA |
| *LOC_Os09g29710* | TGCATCTCTCCTGCAAGAATTA | CAATGACAACCGAAGTGATCAC |
| *LOC_Os01g40870* | GTGACCAAGAACCTCAACATTG | TGGTACATGGTACACTCTAGCT |
| *LOC_Os04g56400* | AGCATTGACAATTTCTCATGGG | CGATTCATCGTCCAAGTTCTTC |
| *Actin* | GAGTATGATGAGTCGGGTCCAG | ACACCAACAATCCCAAACAGAG |
