# Supplementary material for: The Wild Rice Locus CTS-12 Mediates ABA-Dependent Stomatal Opening Modulation to Limit Water Loss Under Severe Chilling Stress
Source: Front Plant Sci. 2020 Oct 30;11:575699. doi: 10.3389/fpls.2020.575699 (PMC7661758; doi:10.3389/fpls.2020.575699)
Supplement: Supplementary file 7 [file Data_Sheet_1.docx]

**The wild rice locus *CTS-12* mediates ABA-dependent stomatal opening modulation to limit water loss under severe chilling stress**

Weijian Cen^1^, Wenlong Zhao^1^, Mingqing Ma^1^, Siyuan Lu^1^, Jianbin Liu^1^, Yaqi Cao^1^, Zhenhua Zeng^1^, Hanxing Wei^1^, Shaokui Wang^3^, Rongbai Li^2#^, Jijing Luo^1#^

Supplementary Material

# Supplementary Figures and Tables

## Supplementary Figures


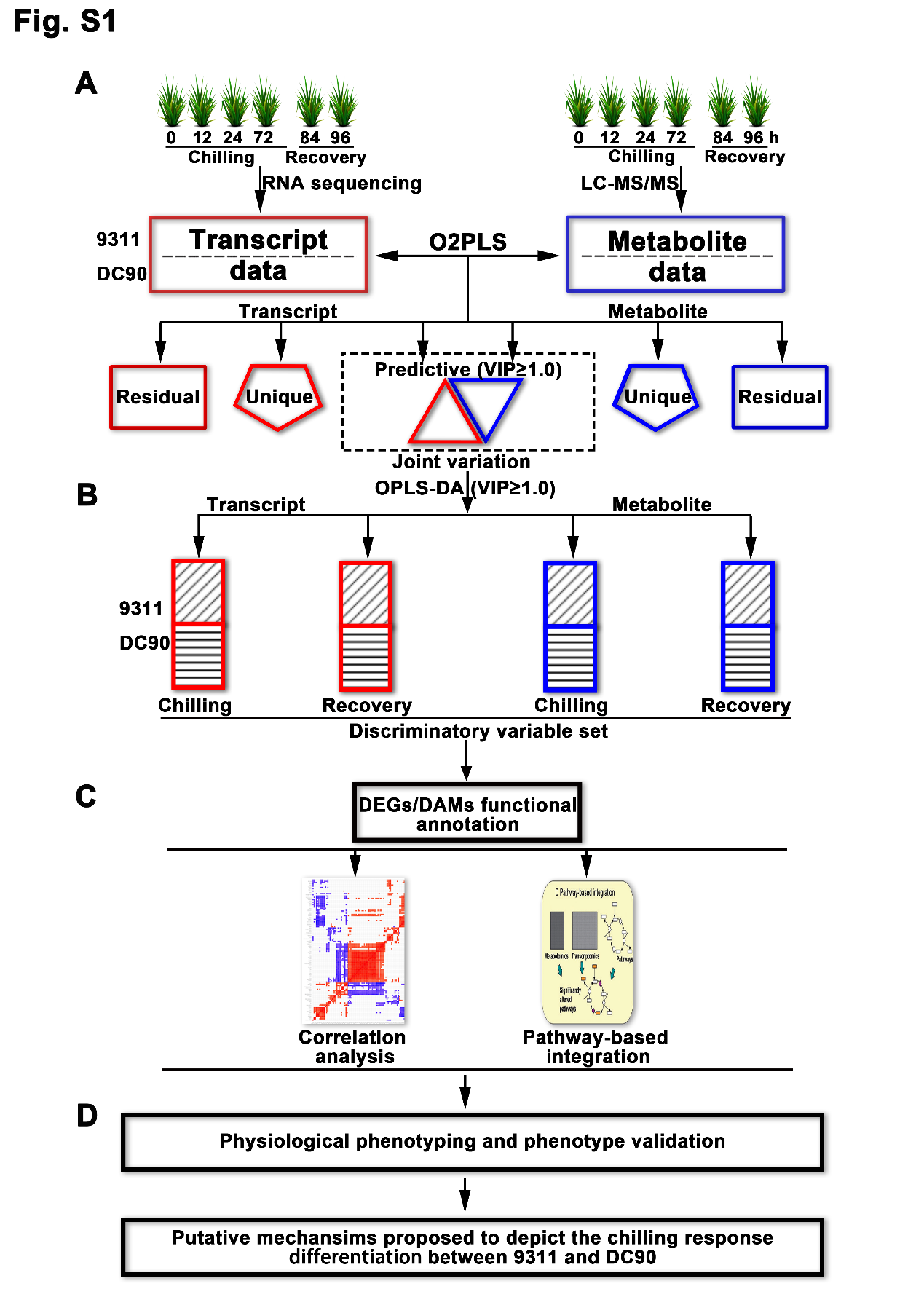


**Figure S1.** Schematic diagram of the experimental design and multivariate data analysis. (A) Sample collection timepoints for LC-MS/MS and RNA-Seq, and O2PLS modeling analysis to identify joint variation variables between transcriptomic and metabolomic data. (B) OPLS-DA modeling analysis to identify variables that differentiate chilling tolerance phenotypes of 9311 and DC90. (C) Functional clustering, correlation analysis, and pathway-based integration of common and discriminatory DEGs/DAMs. (D) Physiological phenotyping, *CTS-12*-mediated phenotype validation, and proposed putative mechanisms underlying the chilling tolerance in rice.


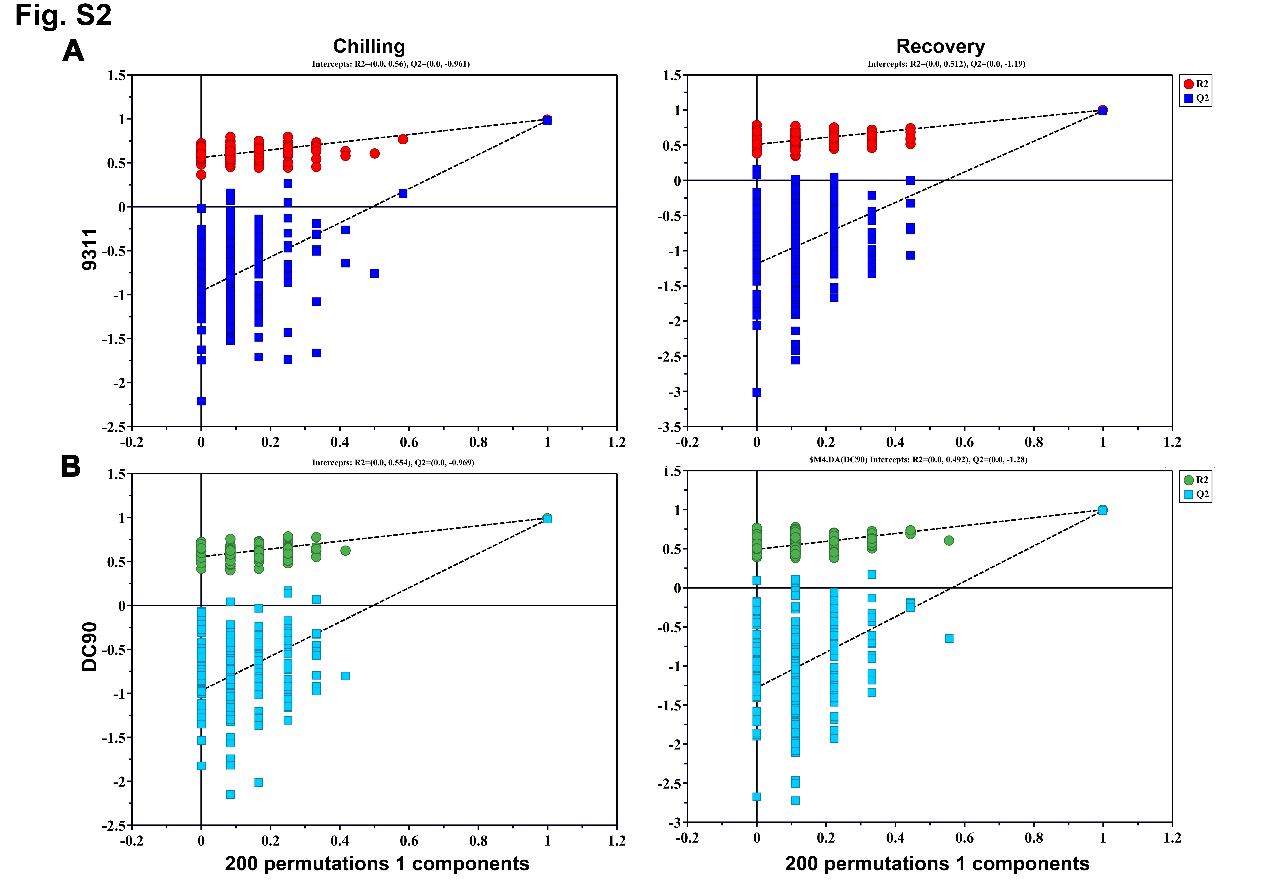


**Supplementary Figures S2.** Permutation test to check the validity and degree of overfit of the OPLS-DA model. (A) The plot displays the validity and degree of overfit of the OPLS-DA model for the 9311 dataset, respectively. (B) The plot displays the validity and degree of overfit of the OPLS-DA model for the DC90 dataset, respectively. The number of permutations is 200. In cross-validation, R2 indicates the fraction of the sum of squares of X or Y explained by the components; Q2 indicates the fraction of the total variation of X or Y that can be predicted by a component.


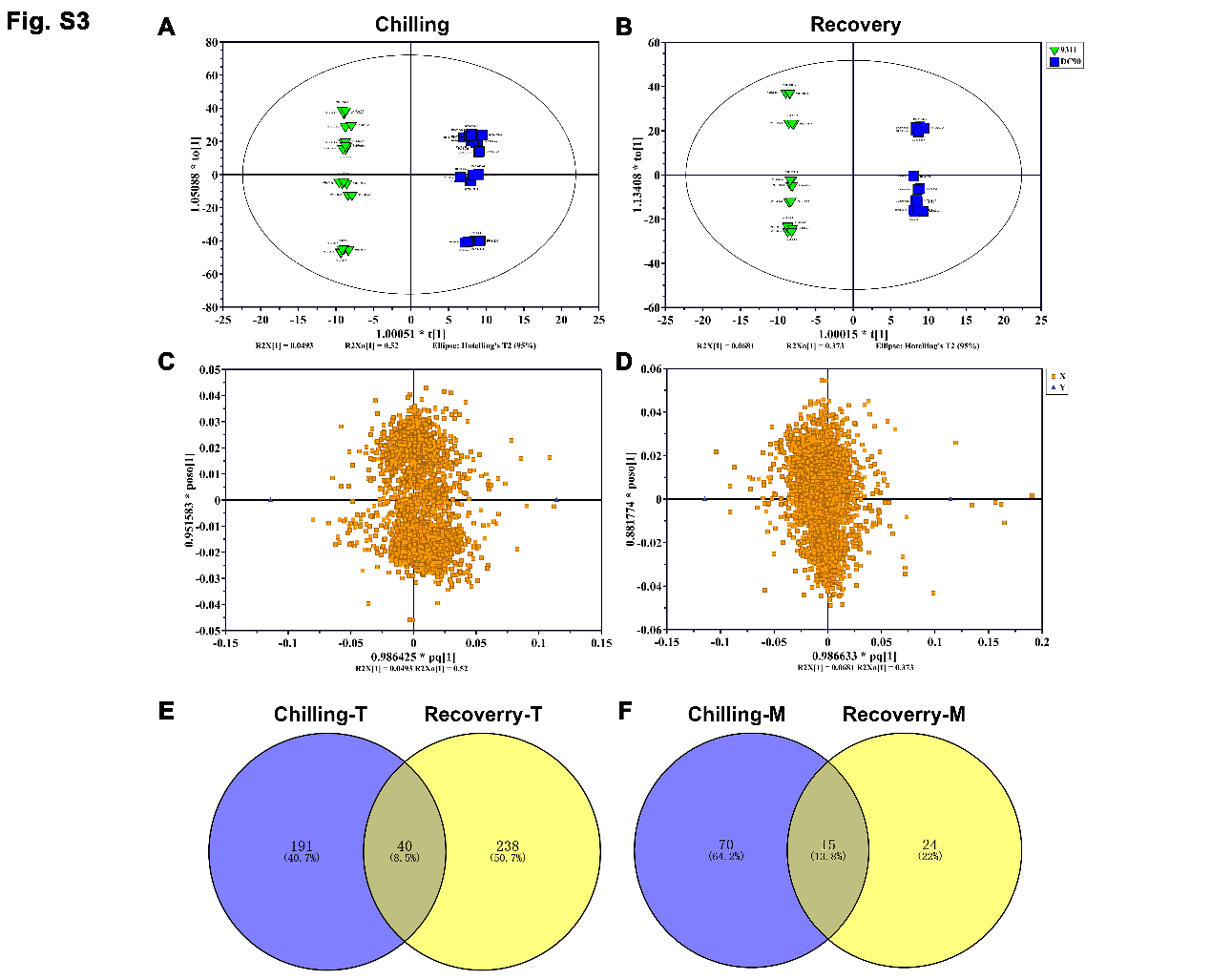


**Supplementary Figures S3.** Identification of the discriminatory variable set differentiating the chilling tolerance phenotypes of 9311 and DC90 by OPLS-DA analysis. (A-B) The score plots of t[1] vs to[1] displaying the separation of 9311 and DC90 in the horizontal (t1) direction, respectively. (C-D) The loading plots of pq[1] vs poso[1] displaying the X-loadings p and the Y-loadings q of the predictive component in the horizontal (pq1) direction, respectively. X-variables situated in the vicinity of the dummy Y-variables have the highest discriminatory power between 9311 and DC90. (E-F) Venn diagram showing the overlap of discriminatory DEGs/DAMs between chilling and recovery treatment. T and M indicate transcriptomic and metabolomic data, respectively.


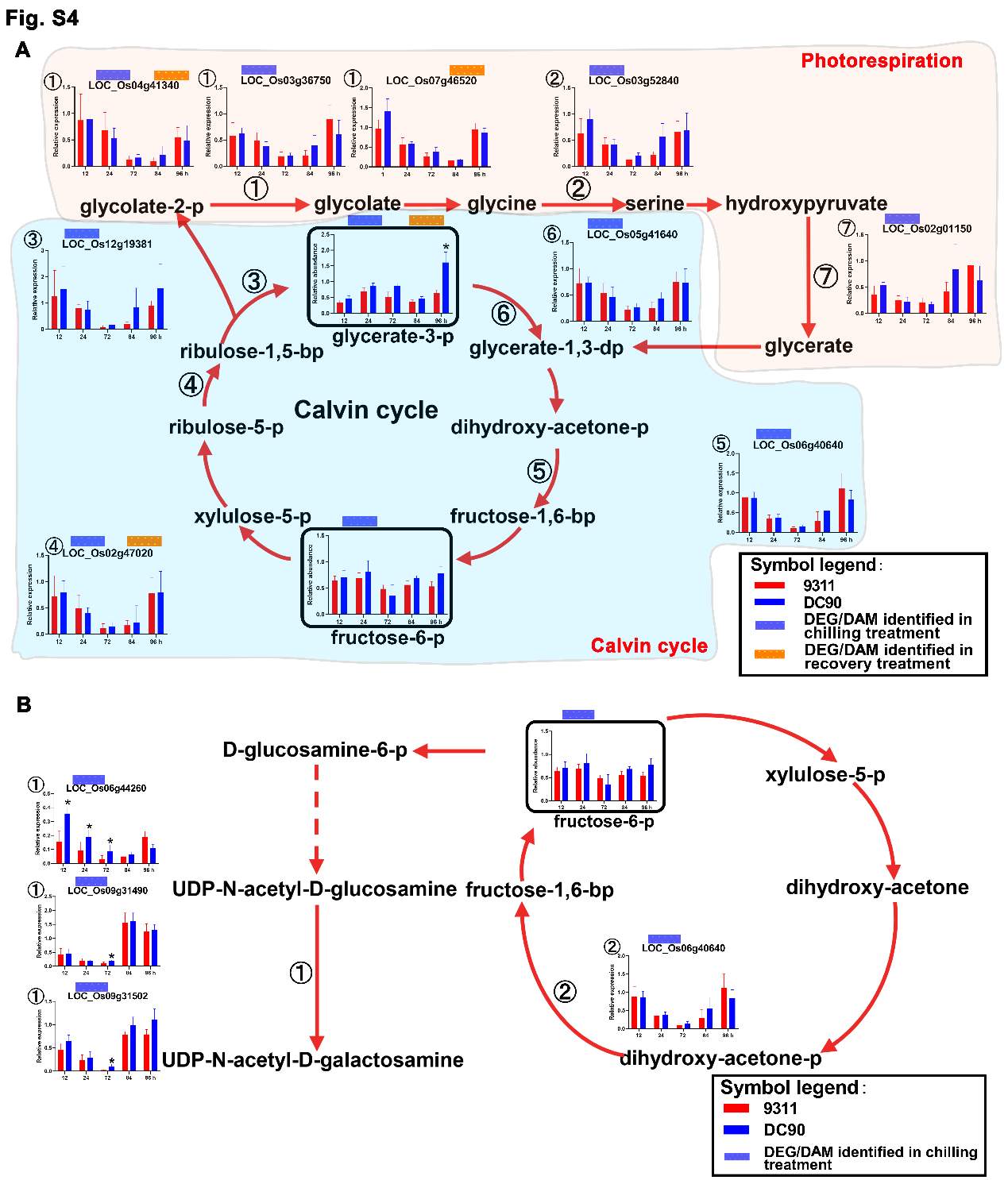


**Supplementary Figures S4.** Chilling- and/or recovery-induced DEGs and DAMs mapped to the Calvin cycle and photorespiration pathways (A) and the UDP-N-acetyl-D-galactosamine biosynthesis pathway (B), respectively. The boxed bar plots in (A and B) represent metabolites with relative abundance alterations at certain timepoints in response to chilling (blue bar at the top) or recovery (orange bar at the top) treatment; Genes encoding related enzymes with relative expression level alteration plots are numbered accordingly; The blue and orange bars at the top of the plot represent significantly altered transcripts or metabolites in response to chilling (blue bar) and/or recovery (orange bar) treatment. The red and dashed arrow in (A and B) indicate overall upstream/downstream relationships and the conversion catalyzed by multiple steps in the related pathways, respectively.


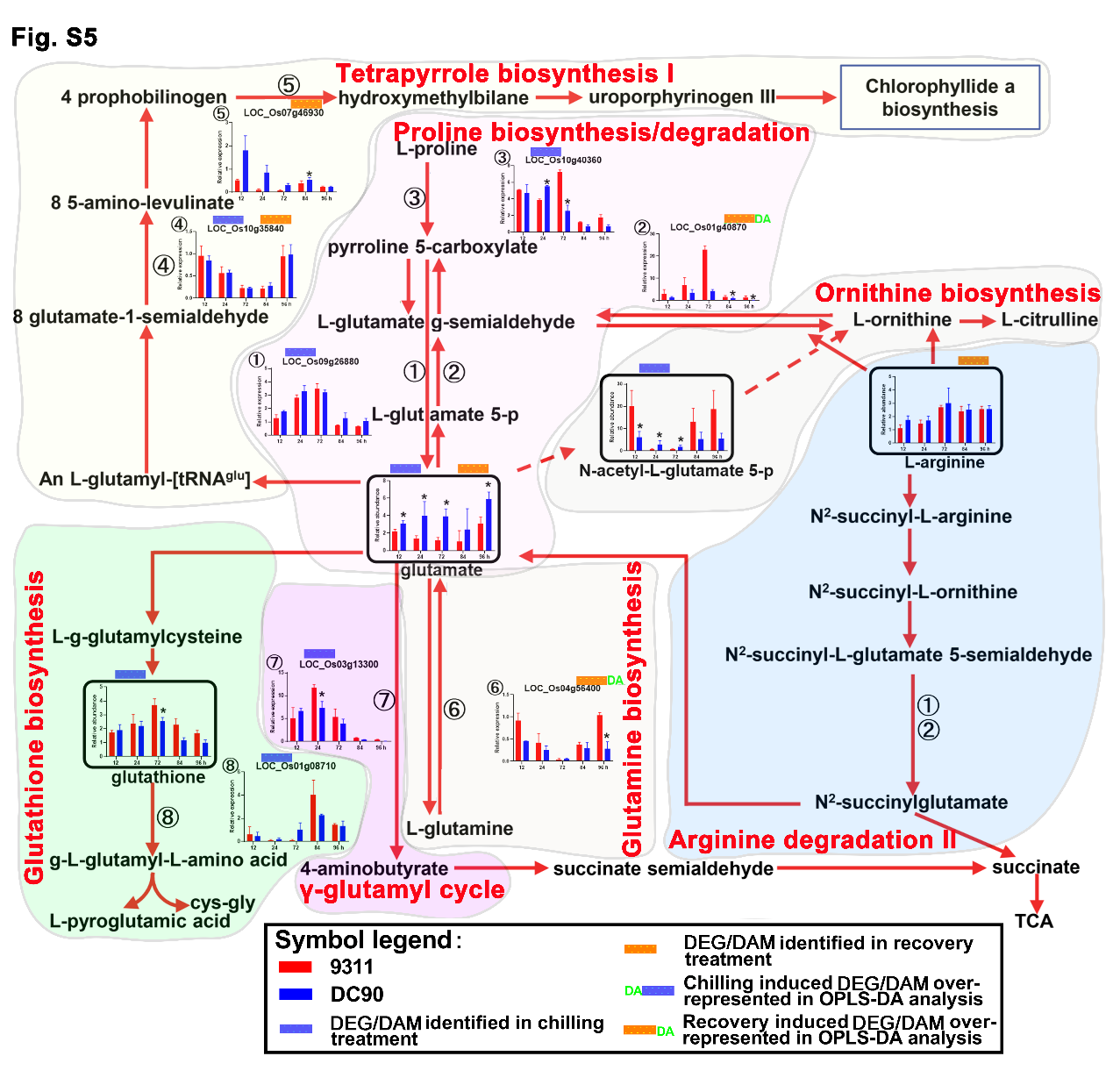


**Supplementary Figures S5.** Chilling- and/or recovery-induced DEGs and DAMs mapped to glutamate-related metabolism pathways. The boxed bar plots represent metabolites with relative abundance alterations at certain timepoints in response to chilling (blue bar at the top) or recovery (orange bar at the top) treatment. Genes encoding related enzymes with relative expression level alteration plots are numbered accordingly. The blue and orange bars at the top of the plot represent significantly altered transcripts or metabolites in response to chilling (blue bar) and/or recovery (orange bar) treatment. The plot of transcripts or metabolites with the green letters DA to the left of the blue bar or to the right of the orange bar represent transcript or metabolite members of discriminatory DEGs/DAMs that play key roles in differentiating the chilling tolerance capacity of 9311 and DC90. The red and dashed arrows indicate overall upstream/downstream relationships and the conversion catalyzed by multiple steps in the related pathways, respectively.


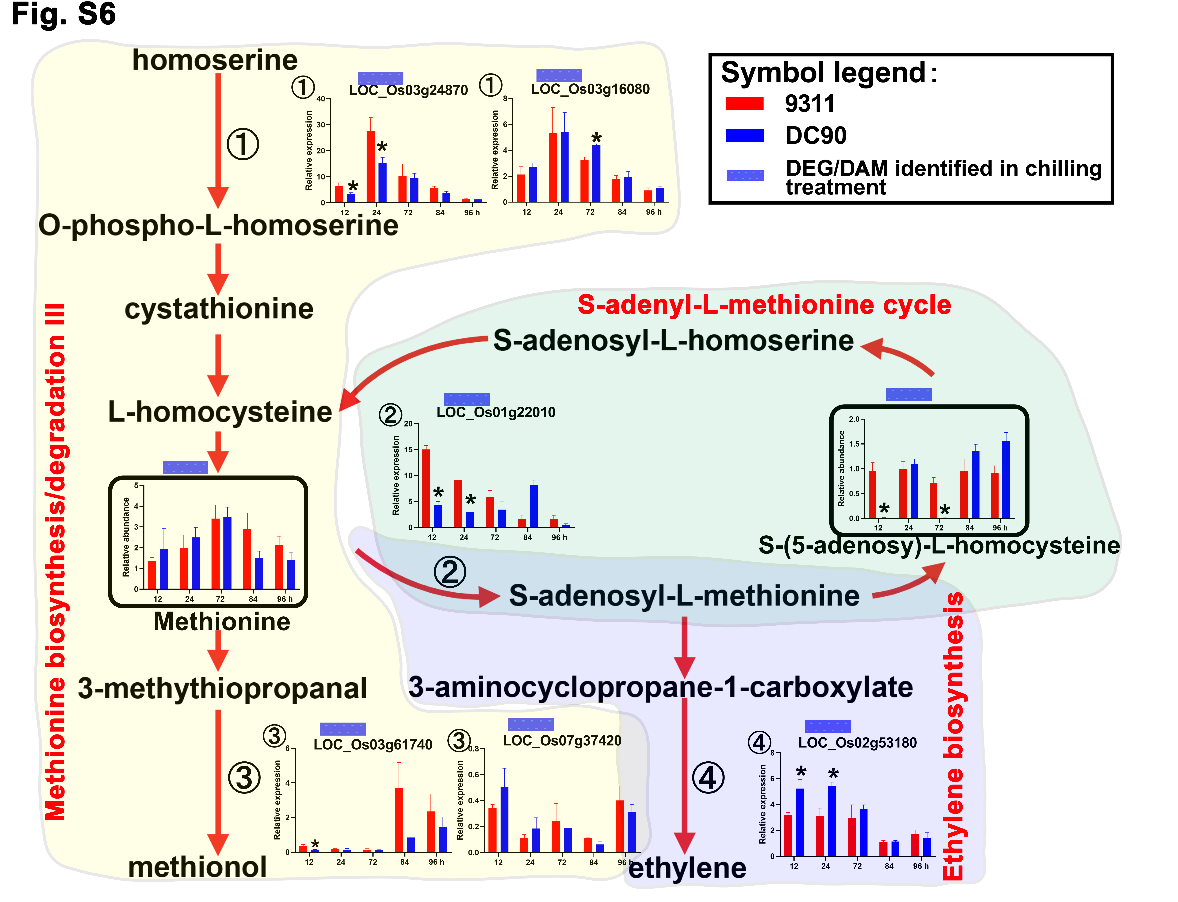


**Supplementary Figures S6.** Chilling-induced DEGs and DAMs mapped to methionine and ethylene metabolic pathways. The boxed bar plots represent the metabolites with relative abundance alterations at certain timepoints in response to chilling (blue bar at the top) treatment. Genes encoding related enzymes with relative expression level alteration plots are numbered accordingly. The blue bar at the top of the plot represents significantly altered transcripts or metabolites in response to chilling treatment. The red arrows indicate overall upstream/downstream relationships in the related pathways.


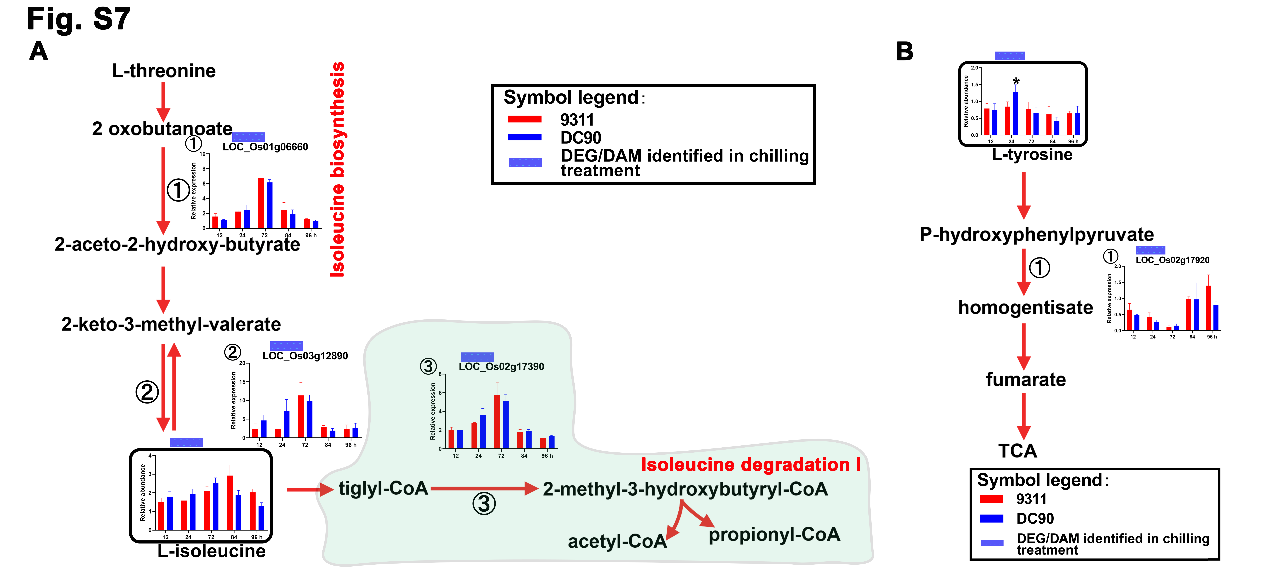


**Supplementary Figures S7.** Chilling-induced DEGs and DAMs mapped to the L-isoleucine metabolic pathway (A) and the tyrosine degradation pathway (B) based on rice metabolic pathways. The boxed bar plots in (A and B) represent metabolites with relative abundance alterations at certain timepoints in response to chilling (blue bar at the top) treatment. Genes encoding related enzymes with relative expression level alteration plots are numbered accordingly. The blue bar at the top of the plot represents significantly altered transcripts or metabolites in response to chilling treatment. The red arrows indicate overall upstream/downstream relationships in the related pathways


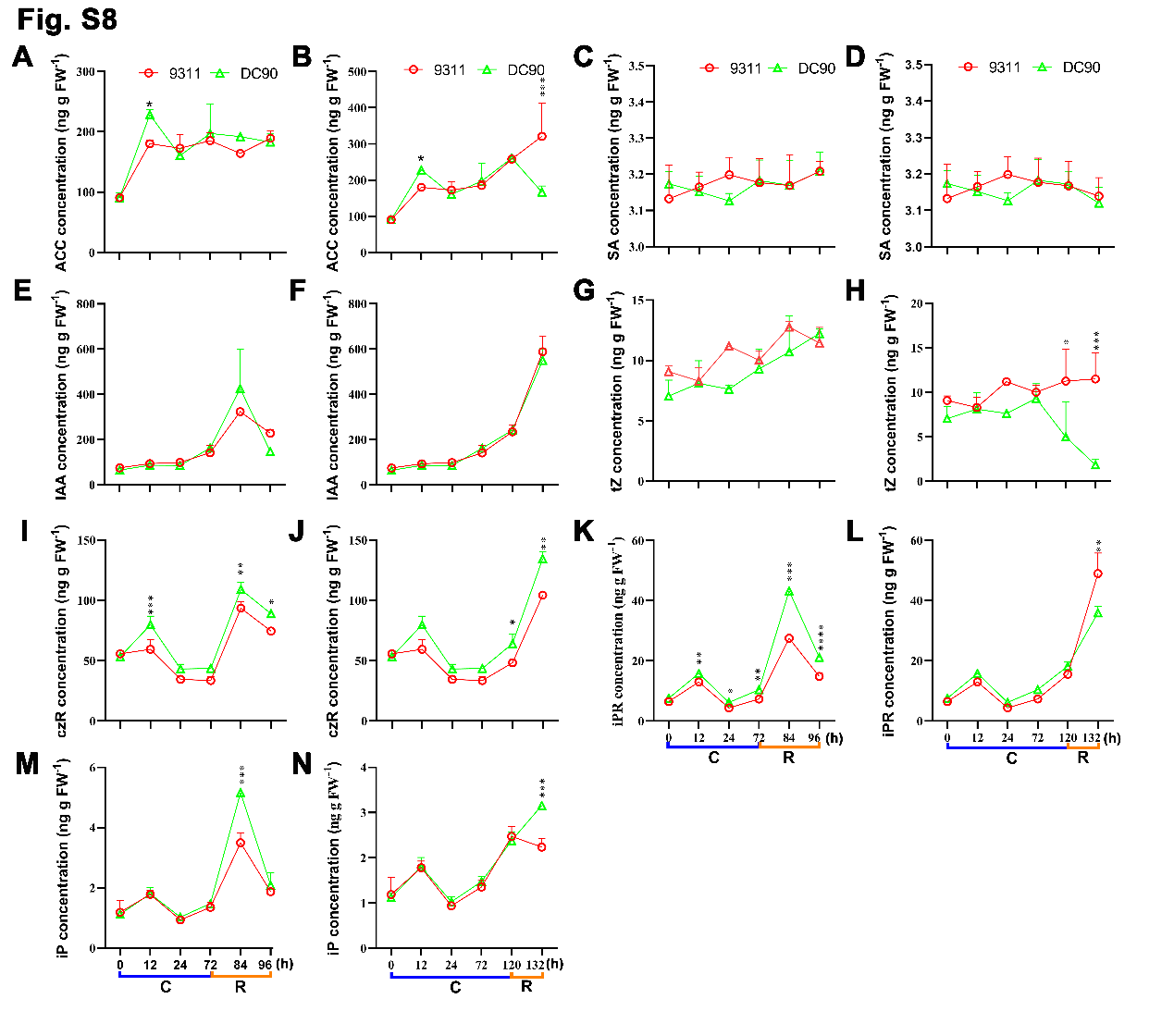


**Supplementary Figures S8.** Targeted quantification of phytohormones in chilling and recovery-treated samples of 9311 and DC90. (A-B) The dynamic changes of ACC in the two treatment regimes, respectively. (C-D) The dynamic changes in salicylic acid (SA) in the two treatment regimes, respectively. (E-F) The dynamic changes in auxin in the two treatment regimes, respectively. (G-N) The dynamic changes in different types of cytokinin in the two treatment regimes, respectively. ‘C’ and ‘R’ in (K-N) designate chilling and recovery periods, respectively.

**Supplementary Figures S9.**


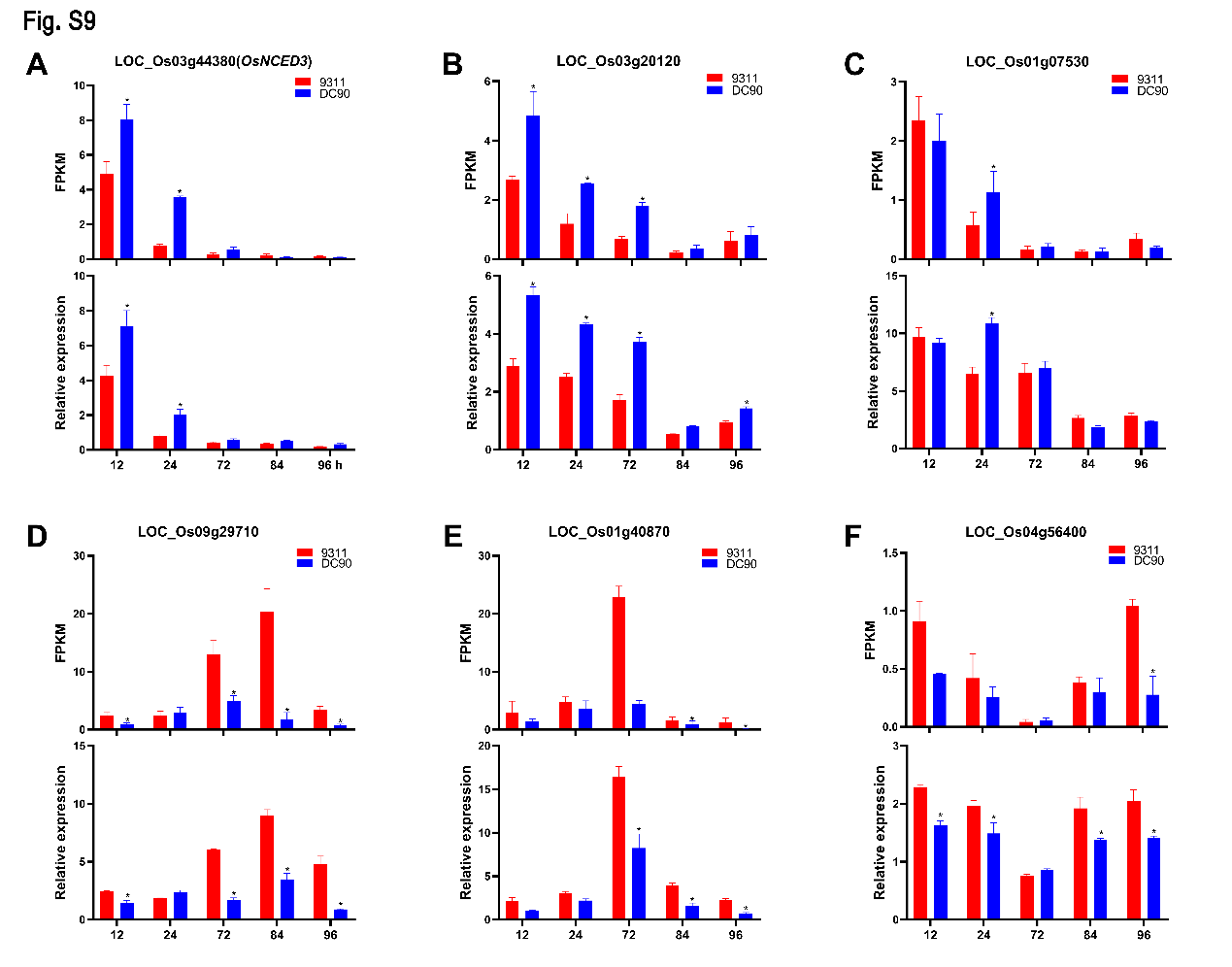


**Supplementary Figures S9.** Validation of the shifts of key discriminatory DEGs identified in the OPLS-DA modelling by qRT-PCR. (A) LOC_Os03g44380. (B) LOC_Os03g20120. (C) LOC_Os01g07530. (D) LOC_Os09g29710. (E) LOC_Os01g40870. (F) LOC_Os04g56400. The sequences of the primers used are shown in Table S6. The up and bottom panels of (A-F) designate the relative expressions of the DEGs in the RNA-seq and qRT-PCR analysis, respectively. FPKM represents Fragments Per Kilobase of exon model per Million mapped fragments


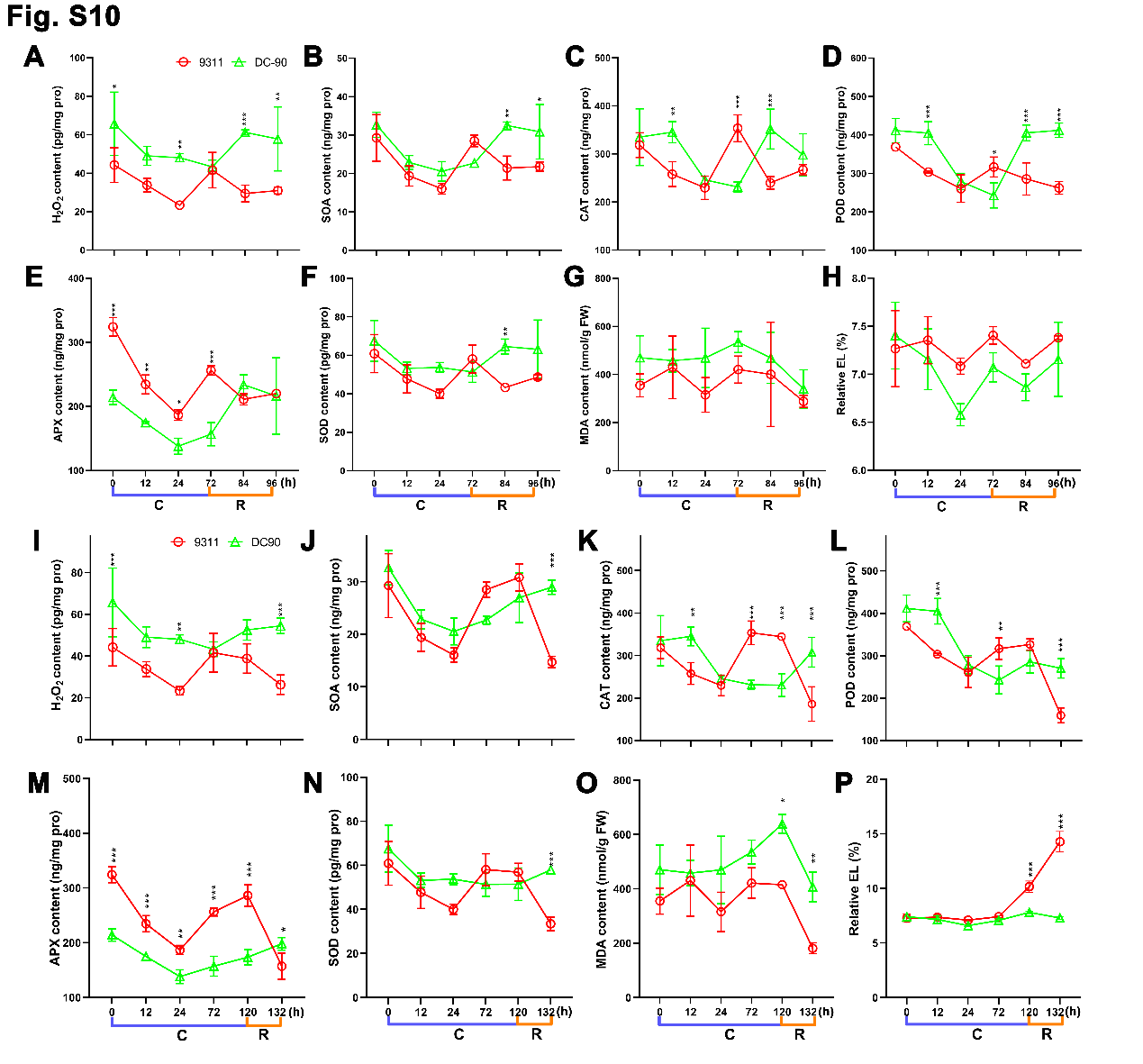


**Supplementary Figures S10.** Examination of ROS production and scavenging in 9311 and DC90 in response to chilling stress and recovery treatment. Line charts showing the production of H_2_O_2_ (A) and superoxide (B) and the activities of catalase (C), peroxidase (D), ascorbate peroxidase (E), and superoxide dismutase (F) in 9311 and DC90. (G) and (H) The production of MDA (F) and relative electrolyte leakage in leaf samples (G), respectively. Data are presented as the mean ± SD. * Significant at *P*≤0.05 and **,***,*** *P*≤0.01 according to Student’s *t* test. ‘C’ and ‘R’ in (E-H; M-P) designate chilling and recovery periods, respectively.


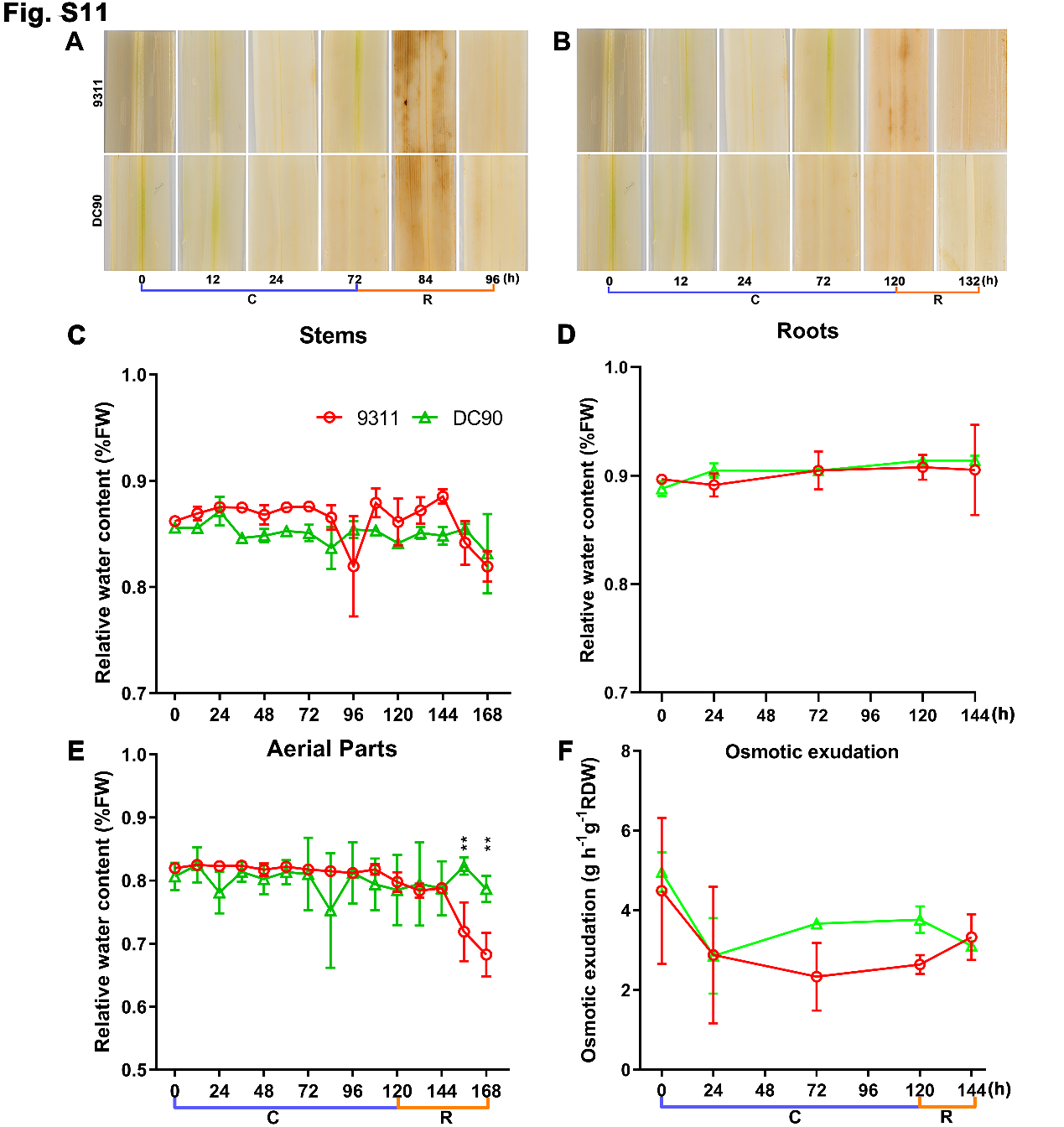


**Supplementary Figures S11** DAB Staining of chilling and recovery-treated leaves and water relation in 9311 and DC90 in response to chilling stress and recovery treatment. (A) DAB-stained leaf samples collected in the 72/24-h treatment regime. (B) DAB-stained leaf samples collected in the 120/12-h treatment regime. (C-E) Line charts showing the water content in stems (C), aerial parts (D), and roots (E) of 9311 and DC90. (F) Osmotic exudation of 9311 and DC90. Data are presented as the mean ± SD. * Significant at *P*≤0.05 and ** *P*≤0.01 according to Student’s *t* test. ‘C’ and ‘R’ in (C-F) designate chilling and recovery periods, respectively.
